# The opposing effects of interferon-beta and oncostatin-M as regulators of cancer stem cell plasticity in triple-negative breast cancer

**DOI:** 10.1186/s13058-019-1136-x

**Published:** 2019-04-29

**Authors:** Mary R. Doherty, Jenny G. Parvani, Ilaria Tamagno, Damian J. Junk, Benjamin L. Bryson, Hyeon Joo Cheon, George R. Stark, Mark W. Jackson

**Affiliations:** 1 Department of Pathology, School of Medicine, Case Western Reserve University, Cleveland, OH 44106 USA; 2 Department of Physiology and Biophysics, University of Illinois at Chicago, Chicago, IL 60612 USA; 3 Case Comprehensive Cancer Center, School of Medicine, Case Western Reserve University, Cleveland, OH 44106 USA; 4 Department of Cancer Biology, the Cleveland Clinic Foundation, Lerner Research Institute, Cleveland, OH 44195 USA

**Keywords:** Interferon-beta, Oncostatin-M, SNAIL, Triple-negative breast cancer, Tumor microenvironment, Cancer stem cells, Cancer stem cell plasticity

## Abstract

**Background:**

Highly aggressive, metastatic and therapeutically resistant triple-negative breast cancers (TNBCs) are often enriched for cancer stem cells (CSC). Cytokines within the breast tumor microenvironment (TME) influence the CSC state by regulating tumor cell differentiation programs. Two prevalent breast TME cytokines are oncostatin-M (OSM) and interferon-β (IFN-β). OSM is a member of the IL-6 family of cytokines and can drive the de-differentiation of TNBC cells to a highly aggressive CSC state. Conversely, IFN-β induces the differentiation of TNBC, resulting in the repression of CSC properties. Here, we assess how these breast TME cytokines influence CSC plasticity and clinical outcome.

**Methods:**

Using transformed human mammary epithelial cell (HMEC) and TNBC cell models, we assessed the CSC markers and properties following exposure to OSM and/or IFN-β. CSC markers included CD24, CD44, and SNAIL; CSC properties included tumor sphere formation, migratory capacity, and tumor initiation.

**Results:**

There are three major findings from our study. First, exposure of purified, non-CSC to IFN-β prevents OSM-mediated CD44 and SNAIL expression and represses tumor sphere formation and migratory capacity. Second, during OSM-induced de-differentiation, OSM represses endogenous *IFN-β* mRNA expression and autocrine/paracrine IFN-β signaling. Restoring IFN-β signaling to OSM-driven CSC re-engages IFN-β-mediated differentiation by repressing OSM/STAT3/SMAD3-mediated SNAIL expression, tumor initiation, and growth. Finally, the therapeutic use of IFN-β to treat OSM-driven tumors significantly suppresses tumor growth.

**Conclusions:**

Our findings suggest that the levels of IFN-β and OSM in TNBC dictate the abundance of cells with a CSC phenotype. Indeed, TNBCs with elevated IFN-β signaling have repressed CSC properties and a better clinical outcome. Conversely, TNBCs with elevated OSM signaling have a worse clinical outcome. Likewise, since OSM suppresses IFN-β expression and signaling, our studies suggest that strategies to limit OSM signaling or activate IFN-β signaling will disengage the de-differentiation programs responsible for the aggressiveness of TNBCs.

**Electronic supplementary material:**

The online version of this article (10.1186/s13058-019-1136-x) contains supplementary material, which is available to authorized users.

## Background

Triple-negative breast cancer is an aggressive subtype that lacks estrogen receptor (ER), progesterone receptor (PR), and amplified HER2. In comparison with other subtypes, TNBC is associated with a higher risk of patient mortality over a 10-year period [1], due in part to the increased development of metastasis and resistance to therapy. These malignant characteristics are attributed to self-renewing cancer stem cells (CSC), which are enriched in TNBC [2]. Identifying novel therapeutic strategies to target CSC remains a critical unmet clinical need. Attractive targets now include cytokines produced by the array of stromal and immune cells within the tumor microenvironment (TME), which are increasingly shown to play important roles in regulating CSC phenotypes [3].

A recent screen of TME cytokines identified oncostatin-M (OSM), a member of the IL-6 superfamily, as a potent inducer of cancer cell de-differentiation, resulting in the acquisition of CSC markers and biological properties (including tumor-initiating capacity, metastatic outgrowth, and drug resistance [2–4]). Following chemotherapy, macrophages at the invasive fronts of tumors secrete elevated levels of OSM [5, 6], and elevated OSM and OSMR correlate with decreased overall survival in patients with TNBC. Mechanistically, OSM activates the heterodimeric receptor complex gp130:OSMR [7], resulting in the activation of JAK/STAT [8] and MAPK signaling pathways. Importantly, we recently demonstrated that OSM-activated STAT3 cooperates with the TGF-β effector SMAD3 to drive increased mesenchymal stem cell properties [4].

In contrast to the CSC-inducing effects of OSM, recent evidence has demonstrated that patients with TNBC harboring elevated numbers of tumor-infiltrating lymphocytes (TILs) and endogenous IFN/signal transducer of activated transcription 1 (STAT1) signaling have an improved therapeutic response and prognosis compared to patients with low TILs and IFN/STAT1 signaling [9–11]. In line with these observations, we recently demonstrated that treatment with IFN-β (a member of the type I IFN family), at a non-cytotoxic/non-cytostatic dose, differentiated CSC into a less aggressive, non-CSC state. Moreover, the elevated expression of an IFN-β metagene signature correlated with repressed expression of a CSC signature and improved patient survival [12]. Mechanistically, IFN-β activates the IFNAR1/2 complex and receptor-associated kinases (JAK1/TYK2) to phosphorylate STAT1 and STAT2, which bind to IRF9 to form phosphorylated interferon-stimulated gene factor 3 (P-ISGF3). This transcription factor drives the expression of hundreds of IFN-stimulated genes (ISGs), including *STAT1*, *STAT2*, and *IRF9*. These three induced proteins form a secondary ISGF3 complex in which STAT1 and STAT2 are not phosphorylated (U-ISGF3), which sustains the expression of a subset of ISGs even in the absence of P-ISGF3. Importantly, we previously demonstrated that IFN-β-mediated CSC differentiation requires robust P-ISGF3 signaling. Unbalanced signaling, resulting in dampened P-ISGF3 but elevated, stable U-ISGF3 expression, promotes rather than represses CSC properties. Likewise, elevated U-ISGF3 has also separately been shown to drive the expression of an IFN-related DNA damage resistance signature (IRDS), which correlates with therapeutic resistance and poor prognosis in a variety of cancers, including breast cancers [13, 14]. Therefore, type I IFN signaling within the TME is a critical determinant of CSC fate and thus clinical outcome.

Here, we show that a non-cytotoxic/non-cytostatic dose of IFN-β, which achieves robust P-ISGF3 signaling, can be used to therapeutically target and repress OSM-mediated CSC properties (including the expression of CSC markers, cell migration, tumor sphere formation, and tumor-initiating capacity). Specifically, IFN-β represses the expression of *SNAIL*, which is driven collaboratively by OSM-activated STAT3, together with the TGF-β effector SMAD3 [4]. SNAIL plays a critical role in driving a mesenchymal/CSC de-differentiation program. Interestingly, we found that OSM opposes IFN-β signaling by repressing endogenous *IFN-β* mRNA expression, thereby inhibiting tumor cell differentiation. Restoring IFN-β signaling effectively opposes OSM-induced de-differentiation. Taken together, our results demonstrate the critical, opposing roles of the TME cytokines IFN-β and OSM in regulating CSC plasticity in TNBC. Our data suggest that maintaining or restoring IFN-β signaling within the breast TME is critical to successfully oppose OSM, which represses endogenous IFN-β expression to undermine the P-ISGF3-mediated induction of ISGs responsible for maintaining cells in a non-aggressive, epithelial, non-CSC state. Collectively, our work suggests that the use of IFN-β can be explored as a potential CSC-targeting therapy for the treatment of aggressive OSM-driven TNBCs.

## Methods

Detailed methods are available in Additional file 1.

## Results

### Sustained IFN-β exposure represses oncostatin-M-mediated CSC properties and inhibits migration

Introduction of transforming elements (shRNAs targeting tumor suppressors p16INK4a and p53 and cDNAs encoding oncogenes c-Myc and H-RAS-V12) to primary human mammary epithelial cells (HMECs) consistently generates two distinct cell populations which can be separated following differential trypsinization: an epithelial/non-CSC (Ep/non-CSC) population and a mesenchymal/CSC (Mes/CSC) population. The Ep/non-CSC population expresses the epithelial proteins E-cadherin and Claudin-1 and a CD24^Hi^/CD44^Lo^ cell surface expression profile characteristic of non-CSC while the Mes/CSC population expresses mesenchymal proteins SNAIL, SLUG, and VIMENTIN, CSC protein NANOG, and a CD24^Lo^/CD44^Hi^ CSC profile and having enhanced migratory capacity and the ability to form tumor spheres. Importantly, the Mes/CSC population has a repressed interferon-stimulated gene (ISG) signature, which can be induced following exposure to recombinant IFN-β. The expression of ISGs following IFN-β treatment occurs concomitantly with the differentiation of Mes/CSC to a less aggressive, epithelial-like state [12, 16]. These findings were clinically validated as elevated expression of an experimentally derived IFN-β metagene signature correlated with repressed expression of CSC-related genes and improved survival outcome in TNBC patients [12].

**Fig. 1:**
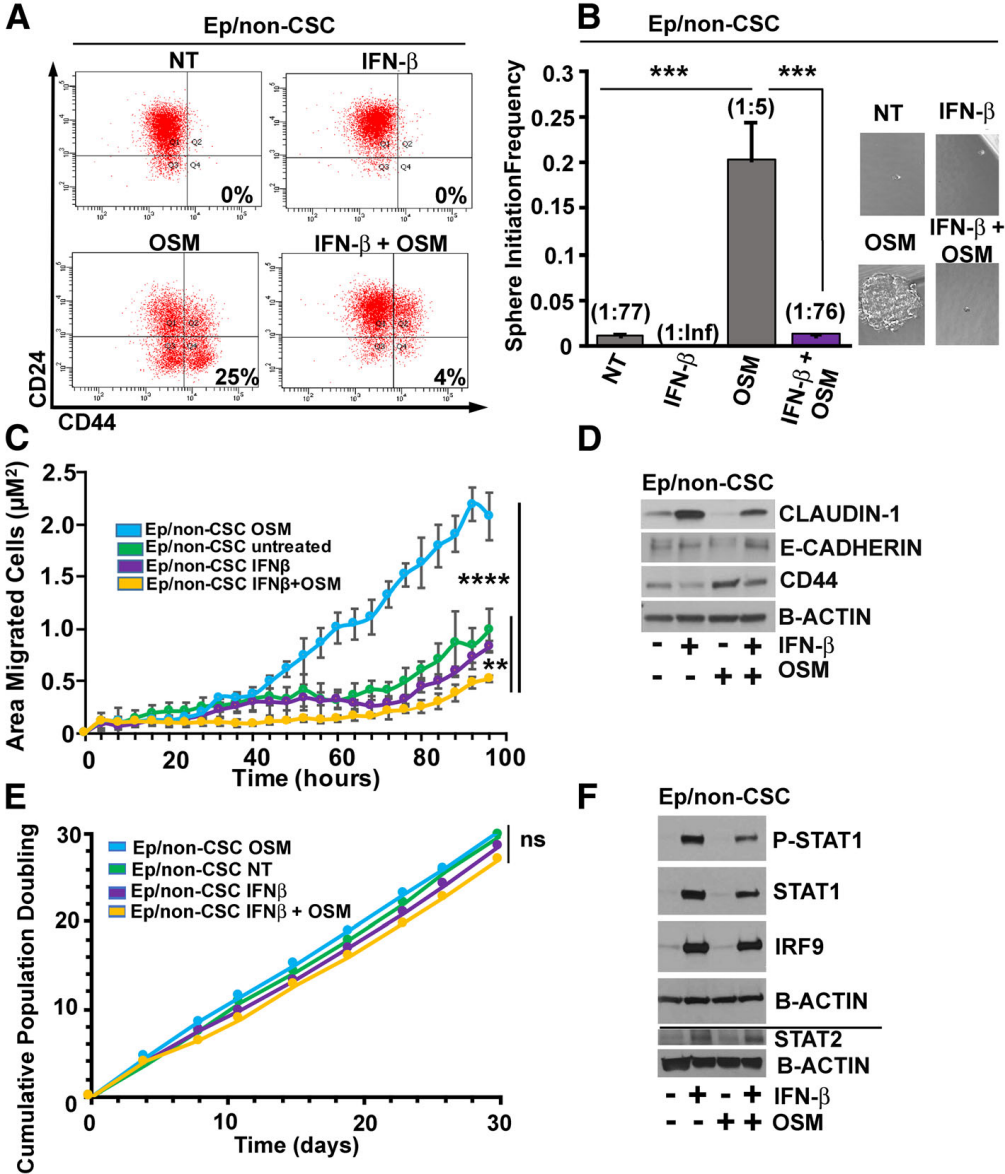
Sustained IFN-β exposure represses oncostatin-M-mediated cancer stem cell properties and inhibits migration. **a** Sustained exposure to IFN-β (Ep/non-CSC pre-treated with IFN-β (100 IU/mL) for 48 h prior to co-treatment with OSM (10 ng/mL) and IFN-β (100 IU/mL); co-treatments for 2 weeks) represses OSM-mediated CD44 acquisition, as shown by flow cytometry (top left, 0% CD44 in NT; top right, 0% CD44 in IFN-β alone; bottom left, 18% CD44 in OSM alone; bottom right, 4% CD44 in IFN-β + OSM co-treatment). **b** Sustained IFN-β exposure (Ep/non-CSC pre-treated with IFN-β (100 IU/mL) for 48 h prior to co-treatment with OSM (10 ng/mL) with IFN-β (100 IU/mL); co-treatments for 3 weeks) significantly represses OSM-mediated tumor sphere initiation at limiting dilution (stem cell frequency: 1:77 for control, 1:Inf (Infinity) for IFN-β, 1:5 for OSM alone, and 1:76 for IFN-β + OSM co-treatment, ***P < 0.0001) ± SD, *n* = 5. **c** Sustained IFN-β (Ep/non-CSC pre-treated with IFN-β (100 IU/mL) for 48 h prior to co-treatment with OSM (10 ng/mL) with IFN-β (100 IU/mL); co-treatments for 4 weeks) followed by removal for 96 h significantly represses OSM-mediated cell migration in Ep/non-CSC (one-way ANOVA, **** *P* < 0.0001) without significantly altering the repressed migration in untreated or IFN-β alone-treated Ep/non-CSC until later time points (one-way ANOVA **P < 0.001, ± SD, 96 h). **d** IFN-β treatment for 48 h followed by co-treatment of IFN-β + OSM for an additional 48 h inhibits OSM-mediated EMT as demonstrated by Western analysis (prevents OSM-mediated repression of Claudin-1 and E-cadherin and inhibits OSM-mediated CD44). **e** Sustained IFN-β exposure (100 IU/mL, every 48 h up to 4 weeks) is non-cytotoxic/non-cytostatic to Ep/non-CSC (one-way ANOVA, ns). **f** Sustained IFN-β exposure (Ep/non-CSC pre-treated with IFN-β (100 IU/mL) for 48 h prior to co-treatment with OSM (10 ng/mL) with IFN-β (100 IU/mL); co-treatments for 3 weeks) maintains canonical IFN-β-signaling mediated through ISGF3 (represented by P-STAT1/STAT1/STAT2/IRF9) signaling alone or in combination with OSM. The line indicates separate Western blots using matched samples

In contrast to IFN-β, exposure of Ep/non-CSC to certain tumor-associated cytokines (such as OSM or TGF-β) can reprogram the cells to a CSC state (with the expression of CSC genes and associated biological activities, including tumor-initiating capacity, invasiveness, and resistance to chemotherapy) [2, 3, 16]. Given these observations, we therefore hypothesized that IFN-β would block the cytokine-mediated reprograming of Ep/non-CSC into Mes/CSC. To test this hypothesis, Ep/non-CSC were pre-treated with IFN-β (100 IU/mL) for 48 h prior to co-treatment with OSM (10 ng/mL), for up to 4 weeks. IFN-β prevented OSM from inducing Mes/CSC properties, as defined by a CD24^Lo^/CD44^Hi^ state (Fig. 1a) and self-renewal capacity (Fig. 1b). In addition, IFN-β prevented OSM-induced migration (Fig. 1c), a phenotype associated with epithelial-to-mesenchymal transition (EMT), a critical component of the metastatic cascade. Examination of mesenchymal markers revealed that IFN-β not only inhibited the OSM-mediated expression of CD44, but also prevented repression of Claudin-1 and E-cadherin (epithelial markers associated with less aggressive breast cancers) (Fig. 1d). Importantly, the impact of IFN-β was not due to a change in proliferation or apoptosis, as 100 IU/mL IFN-β administered every 48 h up to 4 weeks is neither cytostatic or cytotoxic (Fig. 1e). Western analysis confirmed that IFN-β exposure induced canonical signaling through P-ISGF3, demonstrated by robust STAT1 phosphorylation, and upregulated STAT1, STAT2, and IRF9 protein expression (Fig. 1f). In contrast, exposure to type II IFN-γ (1 ng/mL) which signals through the IFNGR1/2 complex and receptor-associated kinases (JAK1/2) to phosphorylate and activate STAT1 homodimers did not repress OSM-mediated CD44 expression, despite driving STAT1 phosphorylation and was neither cytotoxic nor cytostatic (Additional file 1: Figure S1A-C). Taken together, our results demonstrate an important role for IFN-β in repressing OSM-mediated CSC plasticity, further strengthening our prior conclusion that treatment with IFN-β may be a useful therapeutic strategy for repressing the more aggressive, therapeutically resistant features of TNBC tumors.

### IFN-β represses OSM-mediated SNAIL expression

**Fig. 2:**
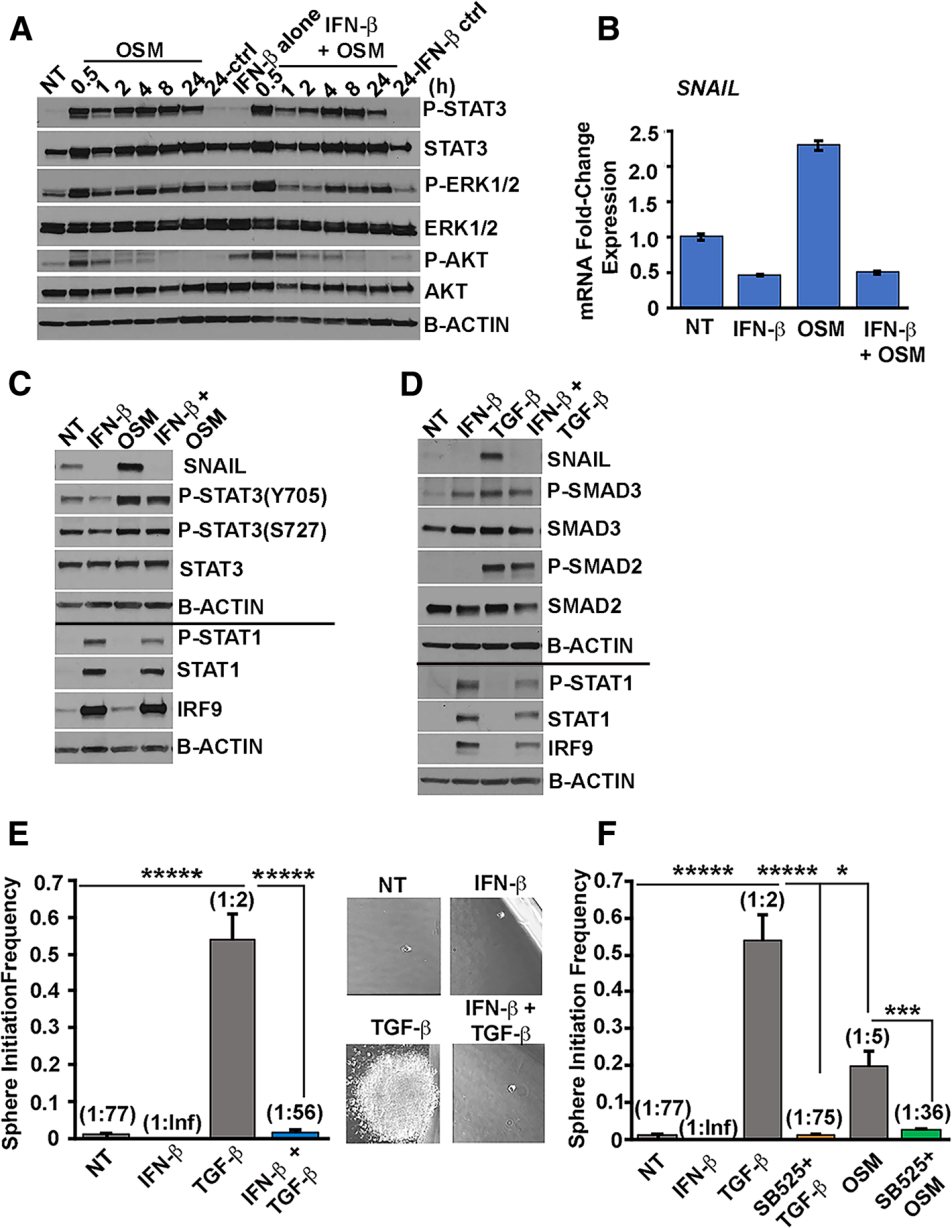
IFN-β represses OSM-mediated SNAIL expression. **a** Acute IFN-β pre-treatment (100 IU/mL, 24 h) does not inhibit the ability of OSM (10 ng/mL, 0.5–24 h) to activate STAT3, MAPK (ERK1/2), and PI3K/AKT via phosphorylation. **b** IFN-β exposure (100 IU/mL, 48 h, followed by IFN-β (100 IU/mL) ± OSM (10 ng/mL) 48 h) significantly represses *SNAIL* mRNA as demonstrated by qRT-PCR and **c** SNAIL protein expression as demonstrated by Western analysis, while retaining robust, canonical IFN-β-mediated signaling (P-STAT1/STAT1/IRF9) signaling. The line indicates separate Western blots using matched samples. **d** IFN-β exposure (100 IU/mL, 48 h, followed by IFN-β (100 IU/mL) ± TGF-β (10 ng/mL), 48 h) significantly represses TGF-β-mediated expression of SNAIL protein with robust IFN-β-mediated signaling (P-STAT1/STAT1/IRF9) signaling. The line indicates separate Western blots using matched samples. **e** Sustained IFN-β (Ep/non-CSC pre-treated with IFN-β (100 IU/mL) for 48 h prior to co-treatment with TGF-β (10 ng/mL) with IFN-β (100 IU/mL); co-treatments for 3 weeks) significantly repressed TGF-β-mediated tumor sphere initiation at limiting dilution (stem cell frequency: from 1:2 TGF-β alone to 1:56 IFN-β+TGF-β; *****P < 0.00001) ± SD, *n* = 5. **f** Pharmacologic repression of TGF-β receptor (Ep/non-CSC pre-treated with TGF-βRI, SB525334, 10 μM for 48 h prior to co-treatment with either TGF-β (10 ng/mL) with SB525334 (10 μM) or OSM (10 ng/mL) with SB525334; co-treatments for 3 weeks) significantly inhibited OSM and TGF-β-mediated tumor sphere initiation at limiting dilution (stem cell frequency: from 1:2 TGF-β alone to 1:75 SB525 + TGF-β; from 1:5 OSM alone to 1:36 SB525 + OSM; *P < 0.05, ***P < 0.001, *****P < 0.0001) ± SD, *n* = 5

We next sought to determine the mechanism by which IFN-β represses OSM-mediated CSC plasticity. Ep/non-CSC were exposed to a single treatment with IFN-β (100 IU/mL, 24 h), followed by OSM (10 ng/mL) for 30 min to 24 h. Western analysis demonstrated that IFN-β did not significantly alter the strength or kinetics of OSM-mediated phosphorylation or expression of the STAT3, ERK1/2, or AKT proteins (Fig. 2a). In line with these findings, IFN-β did not significantly alter the kinetics or levels of suppressor of cytokine signaling 3 (*SOCS3*) mRNA (with only partial repression at 0.5 h post-OSM treatment) (Additional file 1: Figure S2). SOCS3 is a potent negative regulator of STAT3 phosphorylation. We have previously demonstrated that OSM drives Mes/CSC properties through a complex involving STAT3 and the TGF-β effector SMAD3 [4]. Inhibition of TGF-β signaling, using a TGF-β receptor inhibitor or shRNA-mediated ablation of SMAD3, prevented the OSM-mediated induction of Mes/CSC properties. Moreover, we previously identified *SNAIL* as an important STAT3/SMAD3 co-regulated gene [4]. To test whether IFN-β treatment would inhibit OSM-induced expression of *SNAIL*, Ep/non-CSC were treated with IFN-β (100 IU/mL, 48 h) followed by co-treatment with IFN-β (100 IU/mL) and OSM (10 ng/mL) for an additional 48 h. IFN-β significantly repressed the OSM-mediated expression of *SNAIL* mRNA as demonstrated by qRT-PCR (Fig. 2b) and protein (Fig. 2c) as demonstrated by Western analysis. In addition, IFN-β alone repressed basal *SNAIL* mRNA and protein expression, in line with the inhibition of basal tumor sphere formation (Fig. 2b, c and Fig. 1b). In contrast to *SNAIL mRNA*, OSM actually decreased the expression of other mesenchymal- and cancer stemness-associated genes, including *SLUG* and *TWIST*. Although OSM did induce *ZEB1* mRNA, induction of ZEB1 protein was not observed (Additional file 1: Figure S3A-B). IFN-β did not significantly alter OSM’s impact on *SLUG*, *TWIST*, or *ZEB1* mRNA or protein expression (Additional file 1: Figure S4A-D). Importantly, to demonstrate the critical role of SNAIL in driving the Mes/CSC phenotype, we expressed exogenous SNAIL in Ep/non-CSC and confirmed that it induced Mes/CSC properties, as demonstrated by repressed E-cadherin expression, increased CD44 expression, and facilitated tumor sphere formation (Additional file 1: Figure S5A-C). Interestingly, sustained IFN-β treatment (100 IU/mL every 48 h up to 3 weeks) repressed SNAIL-mediated CSC properties including partial reversion of CD44 expression and repressed tumor sphere formation along with partial repression of steady-state SNAIL protein (Additional file 1: Figure S6A-C). Given the suppression of the STAT3/SMAD3 target *SNAIL*, we assessed whether IFN-β could also suppress TGF-β-mediated activation of SNAIL expression and Mes/CSC properties. Indeed, IFN-β strongly inhibited TGF-β-mediated SNAIL protein expression (Fig. 2d) without significantly impacting TGF-β signaling, as judged by the levels of phosphorylated and total SMAD2/SMAD3 (Fig. 2d). Likewise, sustained exposure to IFN-β significantly prevented TGF-β-induced self-renewal, resulting in repressed stem cell frequencies (Fig. 2e). In fact, IFN-β inhibited TGF-β-induced self-renewal with an efficiency similar to that achieved by pharmacologic inhibition of TGF-β (Fig. 2e, f). Our findings demonstrate that IFN-β impinges on a SMAD3/SNAIL axis linking OSM and TGF-β signaling.

### OSM overexpression represses endogenous *IFN-β* mRNA/ISGs and drives mesenchymal/CSC properties in TNBC

**Fig. 3:**
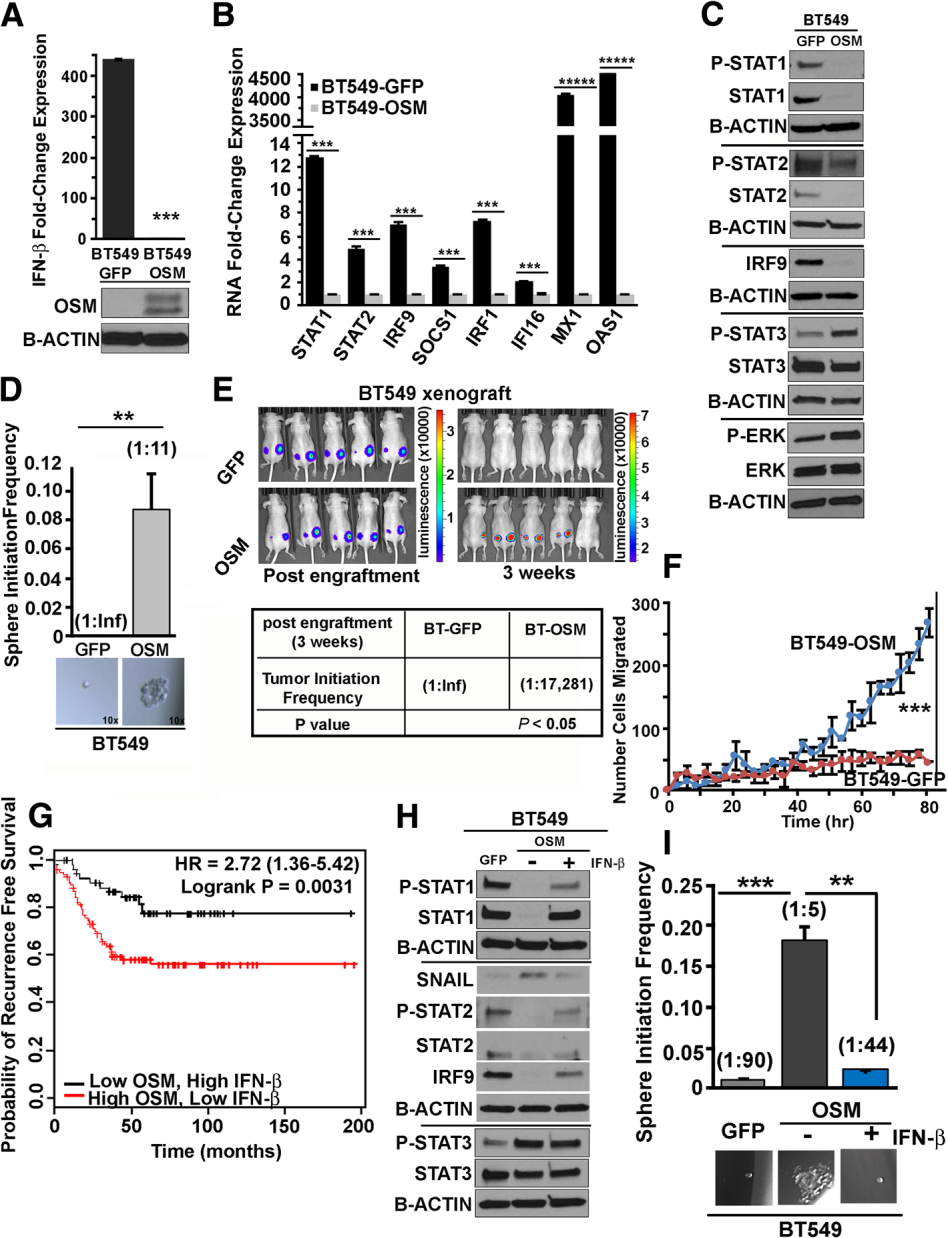
OSM overexpression represses endogenous *IFN-β* mRNA/ISGs and drives mesenchymal/CSC properties in TNBC-BT549 cells. **a** Endogenous *IFN-β* mRNA expression is repressed in BT549-OSM cells, as demonstrated by qRT-PCR (***P < 0.001), ± SEM, *n* = 3. **b** ISGs including *STAT1*, *STAT2*, *IRF9*, *SOCS1*, *IRF1*, *IFI16*, *MX1*, and *OAS1* are repressed in BT549-OSM cells relative to BT549-GFP cells, as demonstrated by qRT-PCR (***P < 0.001, *****P < 0.00001), ± SEM, *n* = 3. **c** IFN-β signaling effectors including phosphorylated and total protein expression of STAT1, STAT2, and IRF9 are repressed in BT549-OSM cells relative to BT549-GFP cells (demonstrating repressed P-ISGF3), while OSM signaling effectors, including the expression of phosphorylated and total STAT3 and ERK1/2, are elevated in BT549-OSM cells, as demonstrated by Western analysis. The lines indicate separate Western blots using matched samples. **d** BT549-OSM cells have significantly increased tumor sphere initiation capacity (stem cell frequencies: 1:11 in BT549-OSM, 1:Inf (Infinity) in BT549-GFP; **P < 0.01), ± SD, *n* = 5. **e** BT549-OSM cells have robust tumor initiation capacity in vivo following 3 weeks of engraftment, relative to BT549-GFP cells (bioluminescent images and table showing tumor initiation frequencies: 1:Inf (Infinity) in BT549-GFP, 1:17,281 in BT549-OSM; *P = 0.05) ± SD, *n* = 5 mice. **f** BT549-OSM cells have enhanced migratory capacity relative to BT549-GFP cells (post-80 h; ***P < 0.001). **g** Elevated expression of an experimentally derived OSM target gene signature (top 20 induced genes) and low expression of an experimentally derived IFN-β target gene signature (top 20 induced genes) corresponds with the decreased patient survival in TNBC (red graph) compared to low expression of the OSM target gene signature and high expression of the IFN-β target gene signature (black graph) (*P* = 0.0031). **h** Sustained exogenous, recombinant IFN-β treatment (100 IU/mL; every 48 h for up to 6 weeks) is sufficient to restore canonical IFN-β-mediated P-ISGF3 signaling, with robust phosphorylation of STAT1 and STAT2 and increased expression of STAT1, STAT2, and IRF9 proteins and repressed expression of SNAIL. The lines indicate separate Western blots using matched samples. **i** Sustained exogenous recombinant IFN-β treatment (100 IU/mL; every 48 h for up to 6 weeks) significantly represses tumor sphere initiation capacity in BT549-OSM cells (stem cell frequencies: 1:90 in BT549-GFP, 1:5 in BT549-OSM, 1:44 in BT549-OSM + rec IFN-β; **P < 0.01; ***P < 0.001) ± SD, *n* = 6

Our recent studies have identified a significant reduction in the ISG signature in cells with Mes/CSC properties and in TNBC tumors with a CSC gene signature [12]. However, it is not clear why the ISG signature is repressed in more aggressive Mes/CSC cells and tumors. When *IFN-β* gene expression was assessed in a panel of TNBC cell lines, high levels were detected in Ep/non-CSC from transformed HMECs as well as in BT549 cells (Additional file 1: Figure S7). In BT549 cells expressing exogenous OSM (BT549-OSM), *IFN-β* and a number of ISGs are significantly repressed when compared to control BT549-GFP cells (Fig. 3a, b). Likewise, OSM induced the repression of ISGs in Ep/non-CSC derived from transformed HMECs, as demonstrated by microarray analysis and validated by qRT-PCR, following 3 weeks of OSM treatment (Additional file 1: Figure S8A-B). Moreover, BT549-OSM cells exhibit decreased P-STAT1 and P-STAT2 levels and decreased STAT1, STAT2, and IRF9 expression, concomitant with increased OSM-induced P-STAT3 and P-ERK activity (Fig. 3c). OSM signaling and the associated repression of *IFN-β* results in an increased tumor sphere frequency in vitro (Fig. 3d), increased tumor-forming capability in vivo (Fig. 3e), and increased migration (Fig. 3f). These malignant characteristics are further validated clinically, whereby TNBC patients harboring high expression of an OSM signature (defined by 20 OSM target genes; Additional file 1: Table S1) and low expression of an IFN-β signature (defined by 20 IFN-β target genes; Additional file 1: Table S2) have significantly reduced survival in comparison with their low OSM signature/high IFN-β signature counterparts (Fig. 3g). Collectively, our data indicate that OSM represses endogenous IFN-β expression, thereby undermining P-ISGF3-mediated induction of ISGs that are responsible for maintaining cancer cells in a non-aggressive, epithelial, non-CSC state.

### Restoration of IFN-β signaling represses OSM-mediated CSC properties and SNAIL expression

**Fig. 4:**
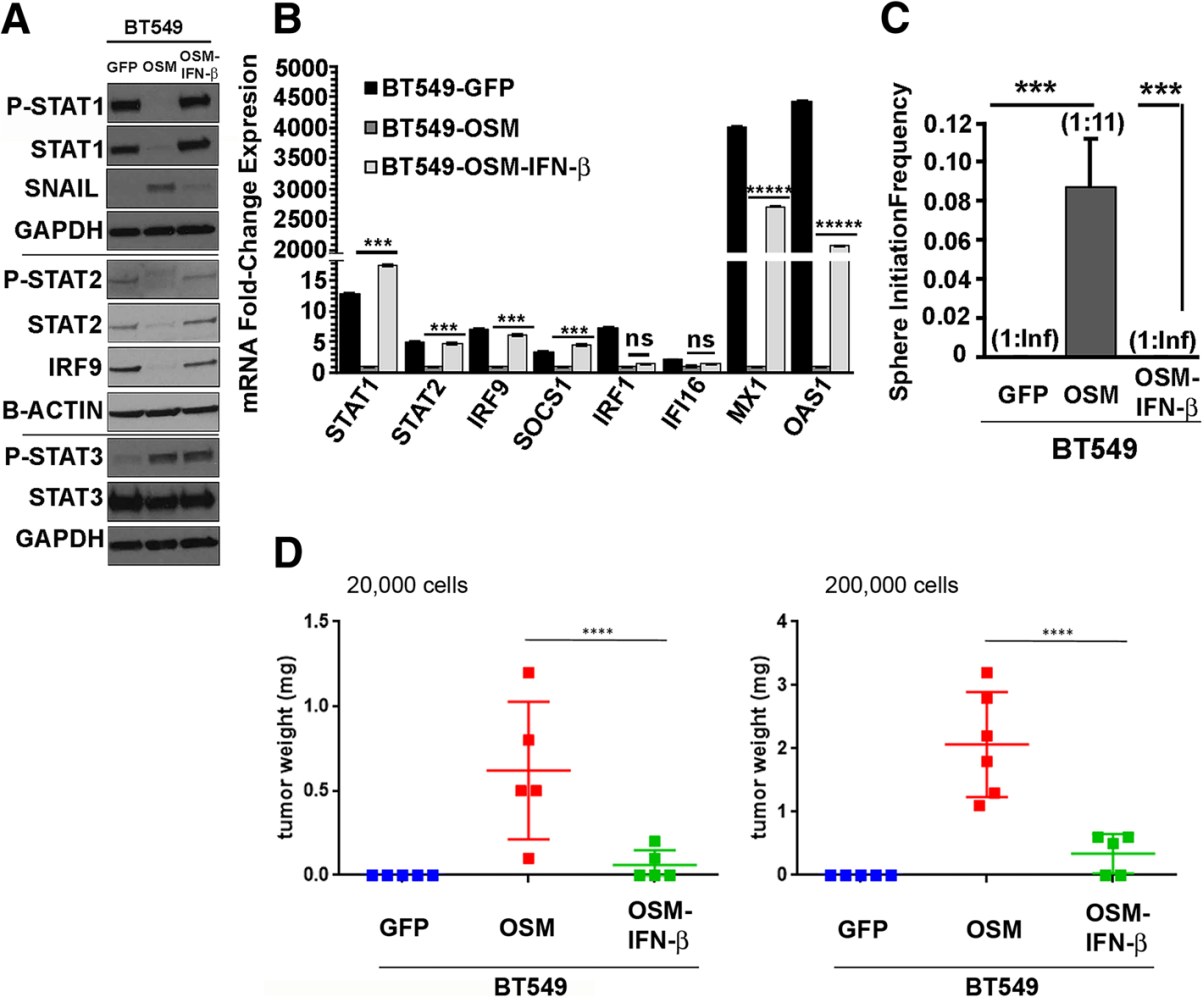
Restoration of IFN-β signaling represses OSM-mediated CSC properties and SNAIL expression. **a** Lentiviral transduction of IFN-β in BT549-OSM cells restores canonical IFN-β signaling comparably to BT549-GFP cells and represses OSM-mediated SNAIL expression independently of STAT3 activation, as demonstrated by Western analysis. The lines indicate separate Western blots were run using matched samples. **b** IFN-β overexpression in BT549-OSM cells restores ISG mRNA expression comparably to BT549-GFP cells, as demonstrated by qRT-PCR (*STAT1*, *STAT2*, *IRF9*, *SOCS1*, *MX1*, *OAS1*) but does not restore *IRF1* and *IFI16* expression (***P < 0.001, *****P < 0.00001) ± SEM, *n* = 3. **c** IFN-β overexpression in BT549-OSM cells significantly represses tumor sphere formation comparably to BT549-GFP (stem cell frequencies: 1:Inf (Infinity) BT549-GFP, 1:11 BT549-OSM, 1:Inf BT549-OSM-IFN-β) (***P < 0.001) ± SD, *n* = 5. **d** IFN-β overexpression significantly represses OSM-mediated tumor initiation in vivo when engrafted subcutaneously at 20,000 or 200,000 cells/injection (nu/nu female mice) as demonstrated by decreased tumor weights at day 21 (end of experiment) (one-way ANOVA, ****P < 0.0001) ± SD, *n* = 5 mice

Next, we asked whether restoring endogenous IFN-β signaling in BT549-OSM cells is sufficient to convert them to a non-CSC state. BT549-OSM cells were treated with recombinant IFN-β (rec IFN-β), which increases the expression of STAT1, STAT2, and IRF9 (Fig. 3h). Again, IFN-β does not alter OSM-mediated STAT3 phosphorylation (Fig. 3h) or expression of the STAT3 target gene *SOCS3* (Additional file 1: Figure S9). In contrast, rec IFN-β was able to repress SNAIL expression (Fig. 3h) and strongly inhibit tumor sphere formation (Fig. 3i). Importantly, as described in the transformed HMEC model, the impact of IFN-β was not due to a change in proliferation, as 100 IU/mL IFN-β is neither cytostatic or cytotoxic (Additional file 1: Figure S10A-B). Alternatively, IFN-β expression was restored from a lentiviral construct encoding a human IFN-β cDNA (Additional file 1: Figure S11). Exogenous over-expression of IFN-β was growth suppressive in control BT549-GFP cells but was well tolerated in BT549-OSM cells and did not alter their growth. IFN-β expression reconstituted downstream effector signaling, resulting in the re-expression of the IFN-β ISGF3 components STAT1, STAT2, and IRF9 and the ISGs *SOCS1*, *MX1*, and *OAS1* (Fig. 4a, b). (The expression of ISGs *IRF1* and *IFI16* was not rescued by IFN-β.) Restoration of IFN-β expression led to the inhibition of SNAIL expression, consistent with the significant inhibition of tumor sphere formation (Fig. 4a, c). Finally, we assessed whether restoration of IFN-β signaling could suppress OSM-driven tumorigenicity. As expected, BT549 cells expressing OSM formed robust tumors, compared to control BT549-GFP cells, which did not form tumors within 21 days even with 200,000 cells/injection (Fig. 4d). In contrast, expression of an IFN-β cDNA in BT459-OSM cells resulted in a significantly less tumor growth, and in some cases, no tumor was detected (Fig. 4d). Taken together, the data show that restoration of IFN-β signaling in cells with elevated OSM signaling (which represses *IFN-β*, downstream effectors, and ISGs) represses SNAIL expression and inhibits tumor sphere frequency in vitro and tumor-initiating capacity in vivo.

### IFN-β treatment suppresses OSM-mediated tumor growth

**Fig. 5:**
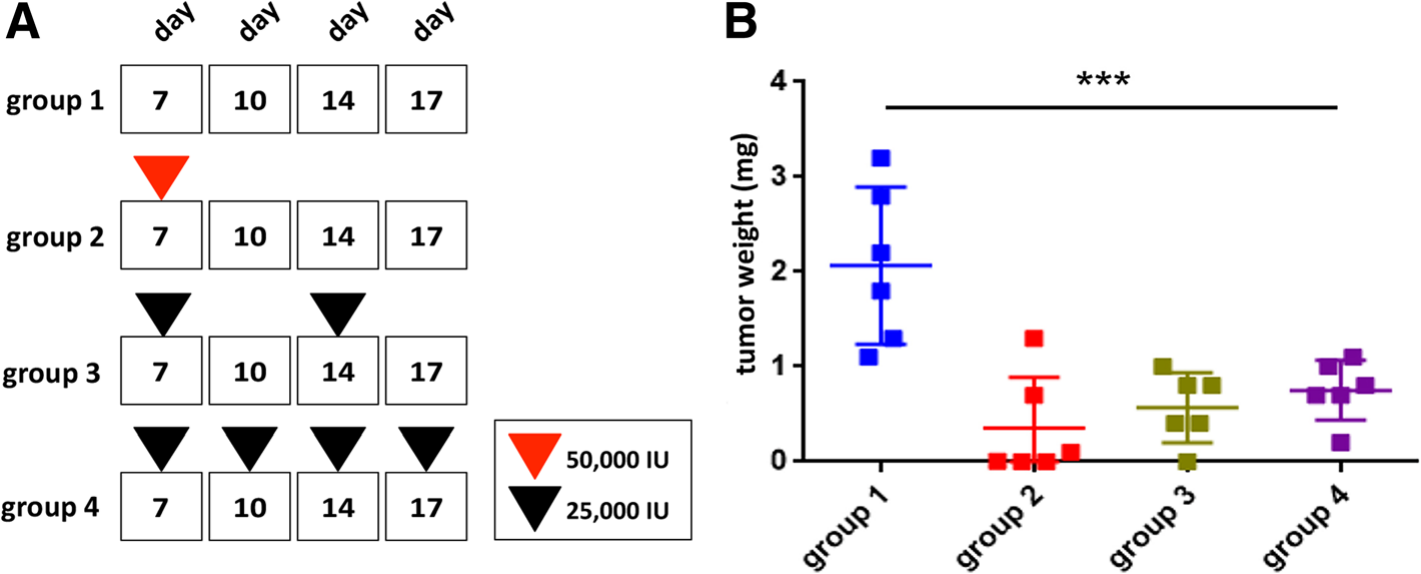
Therapeutic IFN-β represses OSM-mediated tumor growth. **a** Treatment strategy for intra-tumoral administration of recombinant human IFN-β into established BT549-OSM tumors (at 7 days post-engraftment) from top to bottom: group 1 (control no treatment), group 2 (single high dose IFN-β 50,000 IU on day 7), group 3 (25,000 IU administered on day 7 and day 14), and group 4 (25,000 IU administered on days 7, 10, 14, 17). **b** IFN-β treatment significantly reduced OSM-mediated tumor growth under all treatment conditions as demonstrated by repressed tumor weights on day 21 (end of experiment) (one-way ANOVA, ****P < 0.0001) ± SD, *n* = 5 mice

To analyze the therapeutic potential of using IFN-β to suppress CSC properties in vivo, OSM-driven tumors were treated with different IFN-β regimens. On day 7 post-injection, mice with tumors that developed from OSM-expressing BT549 cells were randomized into four treatment groups (five mice each) as follows: untreated (group 1); a single high dose of IFN-β (50,000 IU) on day 7 (group 2); treatments with 25,000 IU of IFN-β on day 7 and day 14 (group 3); and treatments with 25,000 IU of IFN-β on days 7, 10, 14, and 17 (group 4) (Fig. 5a). Three days after the first administration of IFN-β, tumors from all IFN-treated groups began to decrease in size and continued to decrease over time (days 7–21); (Additional file 1: Figure S12). Interestingly, tumors receiving a lower dose of IFN-β more frequently continued decreasing in size; those that received a single higher dose began to re-grow (Additional file 1: Figure S12). At the time of sacrifice (day 21 post-injection), the weights of tumors from all the IFN-β-treated groups were significantly reduced in comparison with those from the untreated group (Fig. 5b). Overall, our in vivo data demonstrate a strong anti-tumor effect of IFN-β treatment. Further experiments will be needed to more thoroughly investigate the mechanisms underlying the results observed and determine the side effects of different treatment schedules on immune-competent animals in order to determine an optimal treatment protocol with IFN-β alone or in combination with other therapeutic options.

## Discussion

TNBC is characterized by therapeutic resistance, metastasis, tumor recurrence, and CSC enrichment [9, 17]. Emerging evidence demonstrates that breast TME plays a critical role in regulating the CSC state. Indeed, we previously demonstrated that cytokines like OSM and TGF-β, which are often elevated in TNBC tumors and portend poor prognosis [3, 6], drive CSC plasticity by de-differentiating less aggressive Ep/non-CSC to highly aggressive Mes/CSC [4, 16]. Importantly, this cytokine-induced CSC plasticity is reversible following cytokine removal or inhibition of downstream effector signaling by genetic disruption or pharmacological blockade [4, 16]. In the current study, we identify how cytokines in the TME can have opposing effects in regulating the differentiation status of TNBC cells. Specifically, we show (i) that IFN-β suppresses the OSM signaling that is responsible for the de-differentiation of non-CSC into CSC and (ii) that OSM can suppress the expression of IFN-β, thereby reducing its ability to maintain cancer cells in a less aggressive, differentiated state. Our studies begin to explain why patients with TNBCs with elevated IFN-β signaling have a better prognosis, as IFN-β can suppress transcriptional programs that confer mesenchymal/CSC properties associated with metastatic outgrowth and therapy failure. We propose that defining and controlling cytokine-mediated CSC plasticity is a clinically relevant therapeutic strategy for treating patients with TNBC.

### IFN-β suppresses pro-CSC cytokine signaling

Using non-cytotoxic/non-cytostatic doses of IFN-β, we saw a significant repression of OSM- and TGF-β-induced stemness (reduced tumor sphere formation, migratory capacity, and a lack of CD44 induction) (Figs. 1 and 2). Interestingly, we did not recapitulate the same anti-CSC effect with type II IFN-γ, which induces phosphorylation of STAT1 (Additional file 1: Figure S1) suggesting that P-STAT1 is insufficient to repress CSC. Rather, IFN-β signaling mediated by ISGF3 signaling (including P-STAT1, P-STAT2, and IRF9) is needed. This observation is in line with our prior findings where we demonstrated that IFN-β but not IFN-γ was sufficient to induce differentiation of CSC into less aggressive non-CSC (evidenced by a robust ISGF3 signaling, requisition of CD24 expression, repressed migration, and repressed tumor sphere formation) [12]. The ability of IFN-β to block OSM- and TGF-β-mediated stemness was not due to the suppression of gp130/OSMR or TGF-βR effectors (STAT3, MAPK, PI3K/AKT, or SMADs) (Fig. 2). Moreover, unlike IFN-γ, which has previously been shown to induce the expression of SMAD7 (a negative regulator of TGF-β/SMADs) [18], IFN-β does not impact SMAD7 expression. Our findings therefore suggest that IFN-β impinges on a CSC target(s) activated by OSM and TGF-β that are critical for promoting tumor cell de-differentiation.

We previously showed that OSM-activated STAT3 cooperates with the TGF-β effector SMAD3 to drive a mesenchymal/CSC program [4]. Mechanistically, STAT3 recruits SMAD3 to the *SNAIL* promoter to drive its expression [4], and importantly, elevated levels of SNAIL are sufficient to drive a mesenchymal/CSC phenotype (Additional file 1: Figure S5A-C). SNAIL, which is often elevated in TNBC tumors and portends poor prognosis [19], plays a critical role in the metastatic cascade by driving EMT and by repressing the expression of key epithelial proteins (E-CADHERIN, OCCLUDIN, CYTOKERATIN) while also inducing the expression of mesenchymal proteins (VIMENTIN). Our current work demonstrates that IFN-β suppresses OSM- and TGF-β-driven SNAIL expression and represses the mesenchymal/de-differentiation program.

Yet, while IFN-β represses *SNAIL* expression, the magnitude does not correlate with the level of SNAIL protein. In fact, IFN-β consistently repressed SNAIL protein more robustly than *SNAIL* mRNA, suggesting that either modest repression of *SNAIL* has a greater impact on protein levels or that IFN-β also regulates SNAIL post-transcriptionally. Interestingly, SNAIL is an unstable protein (~ 25-min half-life) but is aberrantly stabilized in TNBC. Recent work [19] demonstrates that IL-6, which shares a common gp130 receptor with OSM, drives robust SNAIL protein expression despite minimal *SNAIL* mRNA induction. Mechanistically, IL-6 induces the deubiquitinase (DUB3), which removes ubiquitin from SNAIL to prevent its proteasomal degradation. Interestingly, in Ep/non-CSC transduced to express constitutive SNAIL, we found that sustained IFNβ treatment repressed SNAIL-mediated CSC properties (partial reversion of CD44 and repressed tumor sphere initiation capacity) while partially reducing steady-state levels of SNAIL protein (Additional file 1: Figure S6A-C). Our findings support a potential role for IFN-β in regulating SNAIL protein stability as a means to disengage a SNAIL-driven mesenchymal/CSC program. Overall, the inverse correlation between IFN-β and SNAIL protein stability and stemness provides a foundation for future studies aimed at using IFN-β as a therapy to reduce the aggressive features of TNBC.

### Pro-CSC cytokine signaling represses IFN-β

Whereas IFN-β suppresses pro-CSC signaling by OSM/STAT3/SMAD3, we also found the converse to be true, OSM signaling strongly suppresses autocrine IFN-β production, leading to repression of IFN-β targets. In OSM-expressing cells, IFN-β repression resulted in a more aggressive CSC phenotype, leading to enhanced tumor sphere formation and tumor-initiating capacity. Conversely, restoring IFN-β expression in OSM-expressing TNBC cells restored the expression of select ISGs (including *STAT1*, *STAT2*, *IRF9*, *SOCS1*, *MX1*, and *OAS1* and reduced tumor sphere formation and tumor-initiating capacity. Yet, some ISGs, including *IRF1* and *IFI16*, remained repressed even after IFN-β signaling was reconstituted, suggesting that the cell state change induced by OSM may epigenetically alter some genes in a manner that inhibits access by the transcriptional machinery, even after the restoration of IFN-β signaling. Alternatively, OSM may also inhibit transcriptional co-activators or drive transcriptional co-repressors that are specific to these genes. Collectively, these studies suggest that combining methods to neutralize OSM activity in combination with restoring IFN-β signaling may further improve the therapeutic efficacy of IFN-β. While the repression of *IFN-β* mRNA by OSM is clear, the mechanism for the repression remains to be defined. Previous studies demonstrate that IFN-β expression is cooperatively regulated by TGF-β/SMAD3 and IRF7 in transformed cells [20]. Specifically, SMAD3 coordinates with transactivation by IRF7 at the IFN-β promoter. We propose that increased OSM signaling allows activated STAT3 to hijack SMAD3 into a STAT3/SMAD3 complex that shifts the equilibrium away from the SMAD3/IRF7 complex, thereby converting SMAD3 from a tumor suppressor, capable of activating IFN-β, into a tumor promoter that activates a mesenchymal/CSC program.

### A new therapeutic strategy: tipping the scales in favor of IFN-β

Virtually, all nucleated cells, including epithelial and endothelial cells as well as a variety of immune cells (natural killer cells, dendritic cells, macrophages, lymphocytes), can produce type I IFN (IFNα/β) [21]. However, immune cells, especially dendritic cells, are the primary producers of type I IFN. Interestingly, chemotherapy also induces IFN [22]. In fact, in TNBC, favorable responses to frontline chemotherapy correlate with robust IFN signaling [22]. In line with these observations, we and others have previously demonstrated that elevated IFN signaling, resulting in the activation of ISGF3 (P-ISGF3), correlates with “hot” tumors harboring elevated numbers of tumor-infiltrating lymphocytes, active immune surveillance, repression of Mes/CSC properties, and improved therapeutic responses and outcomes [9, 12]. Importantly, a weak IFN response, which results in a dampened P-ISGF3 signaling but robust U-ISGF3 signaling, leading to an IFN-related DNA damage resistance signature (IRDS), can correspond to therapeutic resistance and poor outcomes in a variety of cancers [13–15]. Thus, generating robust P-ISGF3 signaling while minimizing the IRDS is critical for achieving an optimum anti-tumorigenic effect.

OSM is also produced by numerous cell types, including cancer cells, macrophages, and adipocytes [5, 6, 23–25]. In contrast to IFN-β expression, elevated OSM expression is associated with poor clinical outcomes [16]. Given the opposing effects of IFN-β and OSM, it will be important to understand why the TME has evolved to harbor specific levels of each cytokine. However, we propose that the levels of each cytokine, and the opposing effects we observe here, ultimately define the overall stemness of a given tumor. Furthermore, because chemotherapy can induce the expression of both OSM and IFN-β, evaluation of the interplay between these cytokines throughout the treatment may help to inform clinicians about the potential benefit of adding IFN-β or OSM-neutralizing antibodies to treatment regimens.

Our combined in vitro and in vivo studies demonstrate the therapeutic potential of using IFN-β to repress OSM-mediated tumor cell migration and tumor-initiating capacity, respectively. However, within the breast TME, it is likely that IFN-β-mediated immune modulation also contributes to the repression of aggressive CSC properties leading to improved patient survival. Indeed, several pre-clinical studies in immune-competent murine models demonstrate that the loss of type I IFN signaling within the breast TME corresponds with significant metastasis and decreased survival. Importantly, restoring type I IFN signaling (systemic administration of type I IFN or forced expression of type I IFN inducers) in mice bearing tumors significantly decreased metastasis and improved survival outcome [10, 27, 28]. Mechanistically, IFN-β increases tumor antigen cross-presentation to activate potent T cell- and NK cell-mediated antitumorigenic effects (repressed tumor growth and metastases) while inhibiting immunosuppressive cells including MDSCs and Tregs. Importantly, these antitumorigenic effects are specifically a type I IFN-dependent immune-mediated response as mice lacking the type I IFN receptor (Ifnar1−/−), or a functional innate/adaptive immune response, or mice in which T cells and NK cells were neutralized developed metastases with increased mortality [10, 27, 28]. In contrast to IFN-β’s anti-tumor immune response, other pre-clinical studies demonstrate that autocrine OSM signaling potentiates tumor cell immune evasion resulting in spontaneous metastasis while genetic or pharmacological inhibition of OSM signaling represses metastasis [29]. Collectively, our work along with others demonstrate that IFN-β signaling within the breast TME is critical for repressing OSM-mediated CSC plasticity and promoting immune-mediated repression of metastatic outgrowth overall suggesting its significant therapeutic potential in TNBC.

Importantly, while I IFNs have been successfully used in the clinic to treat hematological malignancies and some solid tumors (melanoma), its use in the treatment of breast cancer has largely been ineffective due to dose-limiting toxicities. However, these trials have all used IFN-β at high doses, which is required to achieve anti-proliferative effects [21, 26]. Here, we show that non-toxic/non-cytostatic doses of IFN-β are sufficient to maintain cells in a less aggressive, non-CSC state. This observation provides the rationale for using low-dose IFN-β in combination with chemotherapy in immune-competent murine models, to specifically target and prevent de-differentiation, effectively eliminating an escape mechanism that cancer cells can use when confronted with cytotoxic therapy. In an effort to improve the therapeutic efficacy of IFN, several recent pre-clinical studies have employed targeted delivery-based methods to localize IFN to the TME, including mesenchymal stem cells (MSCs) genetically engineered to express IFN-β (MSC-IFN-β) and targeted monoclonal antibodies to EGFR, and conjugated to IFN-β [27, 28]. A newer, more effective targeting method employs nanobodies, which are single-exon peptides of ~ 110 amino acids that can be cloned, manipulated, and easily mass produced [30]. For future studies to be conducted in immune-competent murine models of TNBC, we envision using a nanobody directed against OSMR conjugated with IFN-β, effectively neutralizing the impact of OSM while also increasing the pro-differentiation and immune-modulatory effects of IFN-β.

## Conclusions

We evaluated how the clinically relevant TME cytokines IFN-β and OSM oppose one another to regulate the differentiation status of TNBC cells. TNBC tumors characterized by elevated IFN signaling, following frontline therapy, have improved clinical outcomes compared to TNBC tumors harboring elevated OSM and repressed IFN signaling. Thus, our studies suggest (i) that maintaining or re-engaging IFN signaling within the breast TME is critical to successfully oppose OSM, which represses the endogenous IFN-β signaling that is needed to maintain cells in a differentiated non-CSC state, and (ii) that using IFN-β to control OSM-mediated CSC plasticity is a clinically relevant therapeutic strategy for treating patients with TNBC.

## Additional file

Additional file 1: Supplementary information. (PDF 1290 kb)

## Abbreviations

ANOVA: Analysis of variance
CSC plasticity: Cancer stem cell plasticity
CSC: Cancer stem cells
ELDA: Extreme limiting dilution assay
Ep/non-CSC: Epithelial/non-CSC
HMEC: Human mammary epithelial cells
IFN-β: Interferon-beta
IFN-γ: Interferon-gamma
Inf: Infinity
IRDS: IFN-related DNA damage resistance signature
ISGs: Interferon-stimulated genes
Mes/CSC: Mesenchymal/CSC
OSM: Oncostatin-M
P-ISGF3: Phosphorylated interferon-stimulated gene factor 3
TGF-β: Transforming growth factor-beta
TME: Tumor microenvironment
TNBC: Triple-negative breast cancer
U-ISGF3: Unphosphorylated interferon-stimulated gene factor 3
