# Supplementary material for: The opposing effects of interferon-beta and oncostatin-M as regulators of cancer stem cell plasticity in triple-negative breast cancer
Source: Breast Cancer Res. 2019 Apr 29;21:54. doi: 10.1186/s13058-019-1136-x (PMC6489282; doi:10.1186/s13058-019-1136-x)
Supplement: Supplementary file 1 — Supplementary information. (PDF 1290 kb) [file 13058_2019_1136_MOESM1_ESM.pdf]

Supplementary Information (SI)

Methods

Cytokine Treatments

Short-term

Transformed HMEC Ep/non-CSC were treated with IFN-β (100 IU/mL) for 24 h, followed by treatment with OSM (10 ng/mL) for 0.5-24 h and monitored for impact on OSM-mediated signaling effectors.

Long-term

Transformed HMEC Ep/non-CSC were treated with IFN-β (100 IU/mL) for 48 h, followed by co-treatment with IFN-β (100 IU/mL) and OSM (10 ng/mL) or TGF-β (10 ng/mL) every 48 h, for up to 4 weeks and monitored for impact on OSM-mediated signaling effectors and CSC properties. BT549-TNBC cell lines overexpressing either GFP (control) or OSM were treated with IFN-β (100 IU/mL) every 48 h, for up to 6 weeks and monitored for impact on OSM-mediated signaling effectors and CSC properties.

Cells and Reagents

Human Mammary Epithelial Cells obtained from a reduction mammoplasty were transformed and cultured as previously described (4). Breast cancer cell lines along with cell culture conditions and source are listed in SI Table 3. All cells were negative for mycoplasma (MycoAlert PLUS Mycoplasma Detection Kit (LT07-705). The rationale for using the HMEC transformation model in the current study is that this model is as an effective tool for studying the impact of TME cytokines on cellular plasticity. As this model generates both Epithelial/non-CSC and Mesenchymal/CSC, these populations can be separated and individually assessed for their response to cytokines (1,3). To verify findings obtained in the HMEC model and to further investigate the connection between OSM and IFN-β signaling in TNBC cell line models, BT549 cells were used since they harbor high basal expression of IFN-β which is most similar to the HMEC-Ep/non-CSC. Recombinant Human Interferon Beta 1a, (Hu-IFN-β 1a) was from PBL assay science (# 11410-2) was used at 100 IU/mL. Human recombinant Interferon-gamma (IFN-γ) was from Peprotech (300-02) and was used at 1 ng/mL. Human recombinant Oncostatin-M (rhOSM) was from Dapcel (#OSM01-13) and used at 10ng/mL. Human recombinant Transforming Growth Factor Beta (rhTGF-β) was from Dapcel (#TGFb101-14) was used at 10ng/mL. TGF-β inhibitor SB525334 was from Seleckchem (#S1476) was used at 10 μM.

Cloning Constructs and Transfections

Viral Vector Cloning

pLenti CMV-Neo GFP was described previously (1). Human IFN-β1 (NM_002176.3) cDNA (Geneopeia; Cat. # GC-A0137-B) was sub-cloned from the Gateway PLUS pShuttle entry vector into the Gateway destination vector pLenti CMV-Neo DEST (705-1) (Addgene #17392) using a Gateway LR Clonase II Enzyme Mix (Invitrogen, cat. #11791-100). Snail cDNA was subcloned from pBabe puro Snail (Addgene Plasmid # 23347) into the pCR8/GW/TOPO entry vector using BamHI and EcoRI (NEB Catalog # R3136S and R3101S, respectively) and sequence-verified. pLenti CMV-Puro Snail was then created by LR Clonase II recombination as described above using the pCR8-Snail GW entry vector and the Gateway destination vector pLenti CMV-Puro DEST (w118-1) (Addgene #17452). Both constructs were sequence-verified after cloning into pLenti CMV-Neo DEST, using the following CMV Forward sequencing primer (5'-to-3'): CGCAAATGGGCGGTAGGCGTG.

Virus Production and Infection
Lentiviruses were produced by transfecting HEK 293T cells with lentiviral expression vectors along with the packaging vectors pCMV-dR8.74 and pMD2.G, using Lipofectamine 2000 as described (1). Both pCMV-dR8.74 and pMD2.G were kind gifts from Dr. Didier Trono (University of Geneva, Switzerland). Supernatant media containing lentivirus was collected 48 h post-transfection, filtered using a 0.22 mm filter, then supplemented with 4 μg/mL polybrene (Santa Cruz Biotechnology; cat. # sc-134220) before being used to infect cells in a humidified atmosphere containing 5% CO2 at 37°C. Infected cells were selected using the antibiotic G418 [200 mg/mL] (Sigma; cat. # 4727878001).

OSM and IFN-β Gene Signatures and TNBC Patient Survival Analysis
The list of top 20 OSM-induced target genes (SI Table 1) were derived from our previously published microarray dataset (1) in which transformed HMEC-Ep/non-CSC were treated ± OSM (10 ng/mL) every 48h for 3 weeks. The list of top 20 IFN-β-induced target genes (SI Table 2) were derived from our previously published microarray dataset (3) in which transformed HMEC-Ep/non-CSC were treated ± IFN-β (100 IU/mL) for 96h. Gene expression is represented as Log2 Fold-change (cytokine-treated relative to their respective untreated controls). These experimentally-derived gene signatures were applied to publicly available TCGA and EGA TNBC patient tumor datasets through Breast Kaplan Meier Plotter (2) to assess the probability of recurrence free survival (Fig 3G). For the analysis, IFN-β target genes were inverted relative to OSM target genes.

Microarray and Quantitative real-time PCR (qRT-PCR)
Microarray analysis of our previously published dataset (1) was used o gauge the impact of OSM on basal IFN-β target gene expression. Briefly, Ep/non-CSC were treated +/- OSM (10 ng/mL) every 48h for up to 3 weeks. Fold change gene expression is represented as the difference in Log2 transformed data of OSM treated samples relative to not treated. Validation of microarray for select ISGs was performed with qRT-PCR using RNA from transformed HMEC (Ep/non-CSC) following treatment +/- OSM (10 ng/mL) every 48 h for up to 3 weeks. For qRT-PCR analysis following short-term cytokine treatments, total RNAs from transformed HMEC (Ep/non-CSC) were pre-treated with IFN-β for 24 h or not, followed by treatment or not with OSM (10 ng/mL) for (0.5-24 h) or were pre-treated with IFN-β or not for 48 h, followed by co-treatment or not with OSM (10 ng/mL) and IFN-β (100 IU/mL) for an additional 48 h. For qRT-PCR analysis using the BT549 cell lines, total RNAs were obtained from BT549-GFP, BT549-OSM, and BT549-OSM cells reconstituted to express IFN-β cDNA that were not treated with exogenous cytokine. Total RNA was also obtained from BT549-GFP and BT549-OSM treated with recombinant IFN-β for 0.5-24 h or not with long-term IFN-β treatments every 48 h up to 6 weeks. RNAs were purified and isolated using the RNeasy Mini Kit (Qiagen), with on column DNase1 digestion, as described (1). qRT-PCR primers for *STAT1, STAT2, IRF9, SOCS1, IRF1, OAS1, MX1, IFI16* (4-5), *SNAIL, SOCS3, B-ACTIN* (1), and *IFN-β* (6) were previously described. qRT-PCR primers: *SLUG* 5'-ACACATTAGAACTCACACGGG, 3'-TGGAGAAGGTTTTGGAGCAG, *TWIST* 5'-CCACTGAAAGGAAAGGCATC, 3'-CTATGGTTTTGCAGGCCAGT, *ZEB1* 5'-ACCCTTGAAAGTGATCCAGC, 3'-CATTCCATTTTCTGTCTTCCGC.

Western Analysis
Cells were lysed, proteins isolated, quantified and immunoblotted as previously described (1). Antibodies used were P-STAT1 Tyr701 (58D6), P-STAT3 Tyr705 (D3A7), P-STAT3 Ser727, P-SMAD2 Ser465/467 (E8F3R), P-SMAD3 Ser423/425 (C25A9), phospho-p44/42-ERK1/2 Thr 202/Tyr 204, P-AKT Tyr 473 (D9E), STAT1 (9H2), STAT3 (124H6), SMAD2 (D43B4), SMAD3

(C67H9), ERK 1/2 (137F5), AKT (40D4), SNAIL (C15D3), SLUG (C19G7), ZEB1 (D80D3), TWIST1, ECADHERIN (24E10), VIMENTIN (D21H3) were from Cell Signaling. P-STAT2 Tyr689 (07-224) was from Millipore, STAT2 (A9), IRF9 were from Santa Cruz Biotechnology, B-ACTIN (Pan-actin Ab-5) was from Neomarkers, GAPDH (6C5) was from Calbiochem.

Flow Cytometry and Microscopy
Transformed HMECs (Ep/non-CSC) (1×10^6), were stained with CD24-phycoerythrin (PE), and CD44-allophycocyanin (APC) and analyzed, and bright-field images were captured as previously described (1).

Growth Assays and Population Doubling
HMECs (Ep/non-CSC) were plated ± IFN-γ (1 ng/mL) in triplicate in 6-well plates at 50,000 cells/well for 96 h. BT549-GFP and BT549-OSM cells ± IFN-β (100 IU/mL) were plated at $1x10^6$ cells per 10-cm plate in triplicate for 72 h. Total cell numbers were quantified using a Beckman Coulter counter and average cell number is shown. BT549-GFP and BT549-OSM cells ± IFN-β (100 IU/mL) were plated at $1x10^6$ cells per 10-cm plate and HMEC (Ep/non-CSC) ± IFN-β (100 IU/mL) or ± OSM (10 ng/mL) or co-treatment IFN-β (100IU/mL) + OSM (10ng/mL) were plated at 300,000 cells per 10-cm plate and assayed for their population doubling capacity as previously described (1).

Limiting Dilution Assay (LDA)
Transformed HMECs (Ep/non-CSC) ± IFN-β (100 IU/mL, 3-4 weeks), BT549-GFP, BT549-OSM and BT549-OSM ± FN-β treatment (100 IU/mL; every 48 h for up to 6 weeks) were live-sorted (DAPI 1:1000) at limiting dilutions (1, 5, 10, cells/well) into 96-well Ultra-Low attachment, polystyrene, flat bottom, sterile, clear plates (#3474 Corning-Costar). Cells were fed with media (50 μL/well) 3 times over the course of 14 days and monitored for tumor sphere formation. Stem cell frequency was calculated using an Extreme Limiting Dilution Assay (ELDA) (7).

Cell Migration Assays
Transformed HMECs (500 cells/well) were seeded into 96-well ClearView plates with 8-mm pores (#4852 Essen Bioscience). Plates were incubated and imaged at 10 x magnification at the indicated time points. Analysis was performed using live cell Incucyte Zoom imaging software (Essen BioScience). Cell migration through the pores to the bottom chambers was imaged and quantified.

Statistical Analysis
Values are means ± SEM for qRT-PCR. Data were compared using 2-tailed student t test *P < 0.05, **P < 0.01, ***P < 0.001, ****P < 0.0001, not significant (ns), n = 3. Values are means ± SDs for migration assays. Data were compared using either 2-tailed student t-test or One-Way ANOVA where indicated *P < 0.5, **P < 0.01, ***P < 0.001, ****P < 0.0001, *****P < 0.00001, not significant (ns), n = 3. Values are means ± SDs for growth assays. Data were compared using 2-tailed student t test *P < 0.05, **P < 0.01, ***P < 0.001, ****P < 0.0001, not significant (ns), n = 3. Differences in Population doubling (PD) over time was calculated using One-way ANOVA *P < 0.5, **P < 0.01, ***P < 0.001, ****P < 0.0001, *****P < 0.00001, not significant (ns). Values are means ± SDs for tumor sphere and tumor initiation frequencies. Stem frequencies were calculated using the ELDA software (17) *P < 0.5, **P < 0.01, ***P < 0.001, ****P < 0.0001, *****P < 0.00001, not significant (ns) (n = 5-6 tumor spheres where indicated, n = 5 tumors). Tumor volumes in mice were compared using One-Way ANOVA *P < 0.5, **P < 0.01, ***P < 0.001, ****P < 0.0001, *****P < 0.00001, not significant (ns), n = 5.

In vivo tumor initiation studies

Mice

Athymic nu/nu female mice (6-8 weeks old) were purchased from the Athymic Animal and Xenograft Core of Case Western Reserve University. All procedures were performed in compliance with the Case Western Reserve University Institutional Animal Care and Use Committee (IACUC).

Bioluminescence Imaging (BLI)

Bioluminescence imaging was performed using IVIS Spectrum In Vivo Imaging System (Perkin Elmer). Mice were anesthetized with 3% Isoflurane in 2l/min oxygen and then injected Intraperitoneally (IP) with 150 μL Luciferin (15 mg/mL, GoldBio) 5 min prior to imaging. Mice were then transferred into the IVIS Spectrum where they were kept under general anesthesia throughout the procedure. Imaging was performed bi-weekly for 3 weeks.

Sub-cutaneous injection of OSM and/or IFN-β-overexpressing cells

BT549 cells transduced with GFP followed by control vector (vec) (BT549-GFP-vec), or vectors encoding human OSM, human OSM and IFN-β, or human IFN-β were subcutaneously injected into previously anesthetized nude mice, as described. 2 different cell numbers were injected into the same mouse: **20,000** in the right flank and **200,000** in the left one. Cells were re-suspended in a solution containing 40% culture medium, 50% Matrigel and 10% Luciferin at a final concentration of 400,000 and $4x10^6$/mL respectively so that 50 μL were injected each time. Tumors were measured with a caliper twice weekly.

Intra-tumoral Injection of IFN-β

Mice were anesthetized with 3% Isoflurane in 2l/min oxygen throughout the procedure. Human OSM-overexpressing BT549 cells (BT549-OSM) were re-suspended in a solution containing 40% culture medium, 50% Matrigel and 10% Luciferin at a final concentration of $4x10^6$/mL. 50 μL, equivalent to **200,000** cells, were subcutaneously injected into both right and left flanks. At day 7 post-engraftment, mice were randomized and distributed into 4 experimental groups to receive intra-tumoral injections of recombinant human IFN-β. Group 1 (control no injection), Group 2 (50,000 IU IFN-β, day 7), Group 3 (25,000 IU IFN-β, day 7, day 14), Group 4 (25,000 IU IFN-β, day 7, 10, 14, 17). IFNβ was prepared in sterile 1 X PBS to reach a final volume of 20 μL. Tumor volume was measured by calipers and calculated using the formula: {[long side * (small side)^2]/2}.

Animal sacrifice and tumor excision

On day 21 post-engraftment, mice were sacrificed by CO2 asphyxiation. Tumors were removed, measured by caliper and weighted.

References

1.Junk DJ, Bryson BL, Smigiel JM, Parameswaran N, Bartel CA, Jackson MW. Oncostatin M promotes cancer cell plasticity through cooperative STAT3-SMAD3 signaling. Oncogene. 2017. 36: 4001-13.

2. Gyorffy B, Lanczky A, Eklund AC, Denkert C, Budczies J, Li Q et al. An online survival analysis tool to rapidly assess the effect of 22,277 genes on breast cancer prognosis using microarray data of 1809 patients. Breast Cancer Res Treatment. 2010;123(3):725-31.

3. Doherty MR, Cheon H, Junk DJ, Vinayak S, Varadan V, Telli ML et al. Interferon-beta represses cancer stem cell properties in triple-negative breast cancer. Proc Natl Acad Sci.2017; 114(52):13792-97.

4. Cheon H, Holvey-Bates EG, Schoggins JW, Forster S, Hertzog P, Imanaka N, et al. IFN-β-independent increases in STAT1, STAT2, and IRF9 mediate resistance to viruses and DNA damage. EMBO J. 2013;32:2751-63.

5. Cheon H, Stark GR. Unphosphorylated STAT1 prolongs the expression of interferon-induced immune regulatory genes. Proc Natl Acad Sci. 2009;106:9373-78.

6. Watanabe A, Tatematsu M, Saeki K, Shibata S, Shime H, Yoshimura A, et al. Raftlin is involved in the nucleocapture complex to induce Poly(I:C)-mediated TLR3 activation. Journal Biol Chem. 2011;286(12):10702-11.

7. Hu Y, Smyth GK. ELDA: Extreme limiting dilution analysis for comparing depleted and enriched populations in stem cell and other assays. J Immunol Methods. 2009; 347: 70-78.

Supplementary Figures and Tables

SI Figure 1. Type II IFN does not repress OSM-mediated CD44 expression in HMEC-Ep/non-CSC cells. (A) Sustained treatment with IFN-γ (1 ng/mL, 2 weeks) does not repress OSM-mediated CD44 acquisition in Ep/non-CSC as shown by flow cytometry (Top left: 0% CD44 in NT, top right: 0% CD44 in IFN-γ alone, bottom left: 25% CD44 in OSM alone, bottom right: 25% CD44 in IFN-γ + OSM co-treatment) (B) despite intact IFN-γ signaling (P-STAT1/STAT1) as shown by western analysis. Line on western blot indicates where blot was cut. (C) IFN-γ (1 ng/mL) is non-cytotoxic/non-cytostatic (ns). Error bars represent ± SD, n=3.

SI Figure 2. IFN-β does not alter the kinetics of OSM-mediated *SOCS3* gene expression. (A) Acute IFN-β pre-treatment (100 IU/mL, 24 h) does not inhibit the overall kinetics of OSM (10 ng/mL, 0.5-24 h) induced STAT3-mediated *SOCS3* mRNA expression (with only partial repression observed at 0.5h post OSM treatment). Data were normalized to *B-ACTIN* as loading control. SOCS3 mRNA expression is represented as fold change relative to Not Treated (NT) control. P-values represent comparisons in gene expression between OSM treated verses IFN-β + OSM co-treated samples (*$P < 0.05$). Error bars represent ± SEM, n=3.

SI Figure 3. OSM preferentially induces SNAIL expression over other Mes/CSC transcription factors. (A-B) OSM exposure (10 ng/mL; 48 h) preferentially induces *SNAIL* mRNA transcription and translation compared to *ZEB1* and induces SNAIL protein but does not alter basal ZEB1 protein, while repressing *SLUG* and *TWIST* mRNA expression and translation. Error bars represent ± SEM, n=3.

SI Figure 4. IFN-β does not alter the OSM-mediated impact on SLUG, TWIST, ZEB1 expression. (A) IFN-β (100 IU/mL; 48 h ± OSM (10 ng/mL) with IFN-β (100 IU/mL) additional 48 h) does not significantly alter basal or OSM-mediated *SLUG* or (C) *ZEB1* mRNA expression, (B) but does repress basal *TWIST* expression, although not significantly altering OSM-mediated repression of *TWIST* mRNA expression. (D) IFN-β (100 IU/mL; 48 h ± OSM (10 ng/mL) with IFN-β (100 IU/mL) additional 48 h) represses basal SLUG and TWIST protein expression comparably to OSM (10 ng/mL), alone or in combination with IFN-β (100 IU/mL). (D) IFN-β (100 IU/mL; 48 h ± OSM (10 ng/mL) with IFN-β (100 IU/mL) additional 48h) represses basal ZEB1 protein expression comparably to IFN-β treatment alone, while OSM treatment alone does not alter basal ZEB1 protein expression. Error bars represent ± SEM, n=3.

SI Figure 5. SNAIL overexpression drives robust Mes/CSC properties in HMEC-Ep/non-CSC. (A) SNAIL is robustly expressed in Ep/non-CSC cells following lentiviral transduction and represses E-cadherin, as shown by western analysis relative to control GFP expressing cells and (B) drives CD44 expression relative to control GFP expressing cells (left panel 0% CD44 in GFP, right panel 82% CD44 in SNAIL) as shown by flow cytometry. (C) SNAIL robustly induces tumor sphere formation (stem cell frequencies: 1:Inf (Infinity) in GFP, 1:20 in SNAIL; ***$P < 0.001$). Error bars represent ± SD, n=6)

SI Figure 6. IFN-β treatment represses SNAIL-mediated CSC properties and SNAIL steady-state protein expression in HMEC-Ep/non-CSC.
(A) Flow cytometry demonstrating that IFN-β treatment (100 IU/mL every 48h for 1 week) partially reduces CD44 expression while increasing CD24 expression in Ep/non-CSC cells expressing SNAIL cDNA (left panel 80% CD44, 13% CD24 in SNAIL-NT; right panel 65% CD44, 24% CD24 in SNAIL+IFN-β). (B) Sustained IFN-β treatment (100 IU/mL, every 48h for 3

weeks) represses SNAIL-mediated tumor sphere formation (stem frequencies; 1:20 in SNAIL NT, 1:92 in SNAIL + IFN-β, $^{**}P < 0.01$). Error bars represent ± SD, n=6. (C) Sustained IFN-β treatment (100 IU/mL, every 48h for 3 weeks) partially represses steady-state SNAIL protein expression as shown by Western analysis (in both dark and light film exposures).

SI Figure 7. *IFN-β* mRNA expression is variable in breast cancer cell lines.
Endogenous *IFN-β* mRNA is robustly expressed in HMEC-Ep/non-CSC and BT549-TNBCs, as well as in cell line models of normal breast (HME1, MCF10A). Error bars represent ± SEM, n=3.

SI Figure 8. Sustained OSM exposure represses basal ISGs in HMEC-Ep/non-CSC.
(A) Sustained OSM treatment (10 ng/mL; every 48 h for 3 weeks) significantly represses basal ISG expression in HMEC-Ep/non-CSC cells, as demonstrated by microarray analysis. (B) qRT-PCR validation of select target ISGs from the microarray ($^{***}P < 0.001$, $^{*****}P < 0.00001$). Error bars represent ± SEM, n=3.

SI Figure 9. IFN-β treatment does not alter OSM-mediated *SOCS3* mRNA expression in BT549 cells.
Acute IFN-β treatment (100 IU/mL, 0.5-24 h) or sustained IFN-β treatment (100 IU/mL; Long-term (LT) for 6 weeks) does not significantly impact *SOCS3* mRNA expression in BT549-OSM cells relative to BT549-GFP cells (also treated with IFN-β 100 IU/mL). Error bars represent ± SEM, n=3.

SI Figure 10. Exogenous IFN-β does not impact growth of BT549 cells.
(A) Acute exposure to exogenous, recombinant IFN-β (100 IU/mL, 72 h) does not alter growth of BT549-OSM expressing cells, while BT549-OSM cells proliferate more rapidly than BT549-GFP cells, which express endogenous IFN-β (ns, $^{**}P < 0.01$ respectively). Error bars represent ± SD, n = 3. (B) Sustained exogenous, recombinant IFN-β treatment (100 IU/mL; every 48 h for 55 days) does not alter proliferation of BT549-OSM cells, which have significantly increased population doubling times compared to BT549-GFP cells which express endogenous IFN-β (one-way ANOVA, $^{***}P < 0.001$, post d 24).

SI Figure 11. Lentiviral-mediated restoration of *IFN-β* mRNA expression in BT549-OSM cells. *IFN-β* mRNA expression is restored in BT549-OSM cells following lentiviral transduction and exceeds the basal endogenous *IFN-β* mRNA levels of BT549-GFP cells. Both BT-GFP and BT-OSM-IFN-β cells have significantly elevated IFN-β mRNA expression, relative to BT-OSM cells ($^{***}P < 0.001$). Error bars represent ± SEM, n=3.

SI Figure 12. Intra-tumoral treatments with IFN-β repress tumor growth over time in BT549-OSM expressing tumors.
IFN-β intra-tumoral administration beginning on Day 7 post tumor cell injection (designated by arrow) decreases tumor size over-time among all IFN-β treatment groups relative to untreated controls (group 1 untreated controls represented by blue graph; group 2 single high dose IFN-β (50,000 IU) on day 7 represented by red graph, group 3 (25,000 IU IFN-β) on day 7, 14 represented by yellow graph; group 4 (25,000 IU IFN-β) on day 7, 14, 17 represented by purple graph. (One-Way Anova $^{***}P< 0.001$). Error bars represent ± SD, n=3.

SI Figure 1

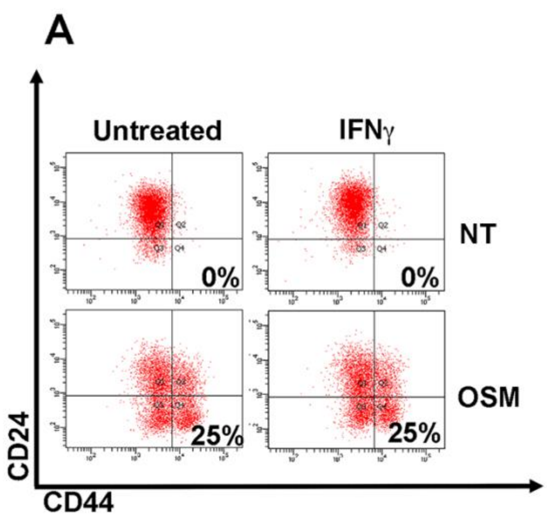
A
Untreated
IFNγ
NT
0%
0%
OSM
25%
25%
CD24
CD44
B
Untreated
IFNγ
P-STAT1
STAT1
GAPDH
C
ns
Number of Cells
(x 100,000)
16
14
12
10
8
6
4
2
0
NT
IFN-γ

SI Figure 2

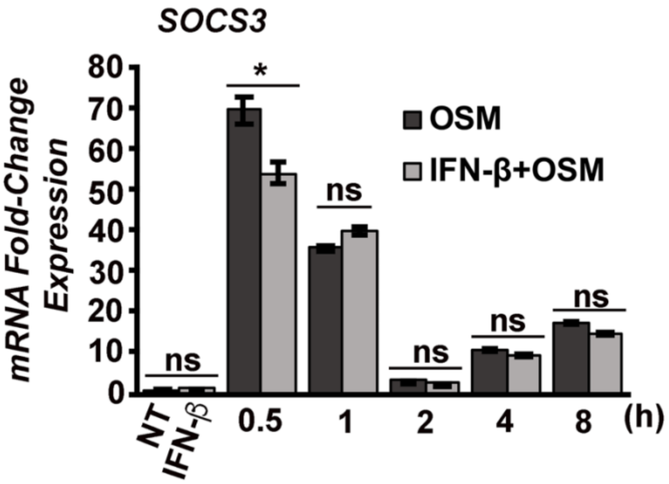
SOCS3
mRNA Fold-Change Expression
80
70
60
50
40
30
20
10
0
*
ns
ns
ns
ns
ns
OSM
IFN-β+OSM
NT
IFN-β
0.5
1
2
4
8
(h)

SI Figure 3

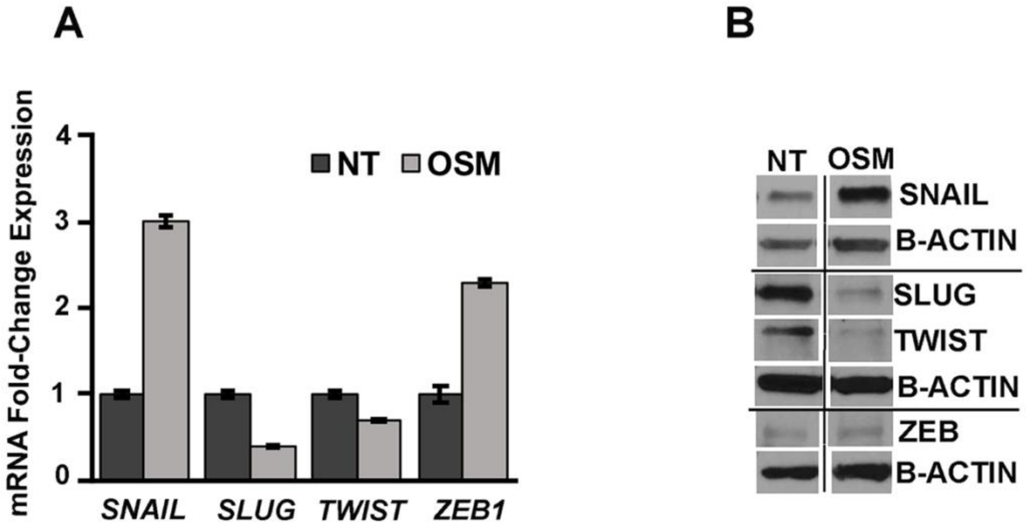
A
B
mRNA Fold-Change Expression
4
3
2
1
0
NT
OSM
SNAIL
SLUG
TWIST
ZEB1
NT
OSM
SNAIL
B-ACTIN
SLUG
TWIST
B-ACTIN
ZEB
B-ACTIN

SI Figure 4

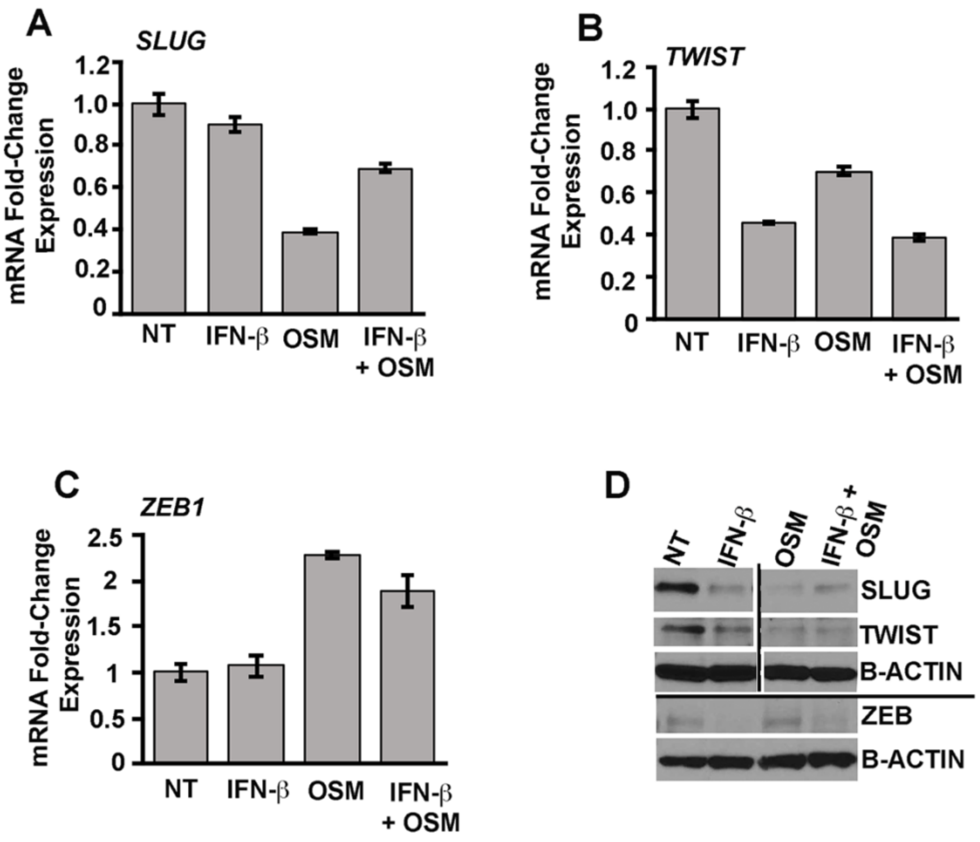
A
SLUG
mRNA Fold-Change Expression
1.2
1.0
0.8
0.6
0.4
0.2
0
NT
IFN-β
OSM
IFN-β + OSM
B
TWIST
mRNA Fold-Change Expression
1.2
1.0
0.8
0.6
0.4
0.2
0
NT
IFN-β
OSM
IFN-β + OSM
C
ZEB1
mRNA Fold-Change Expression
2.5
2
1.5
1
0.5
0
NT
IFN-β
OSM
IFN-β + OSM
D
NT
IFN-β
OSM
IFN-β + OSM
SLUG
TWIST
B-ACTIN
ZEB
B-ACTIN

SI Figure 5

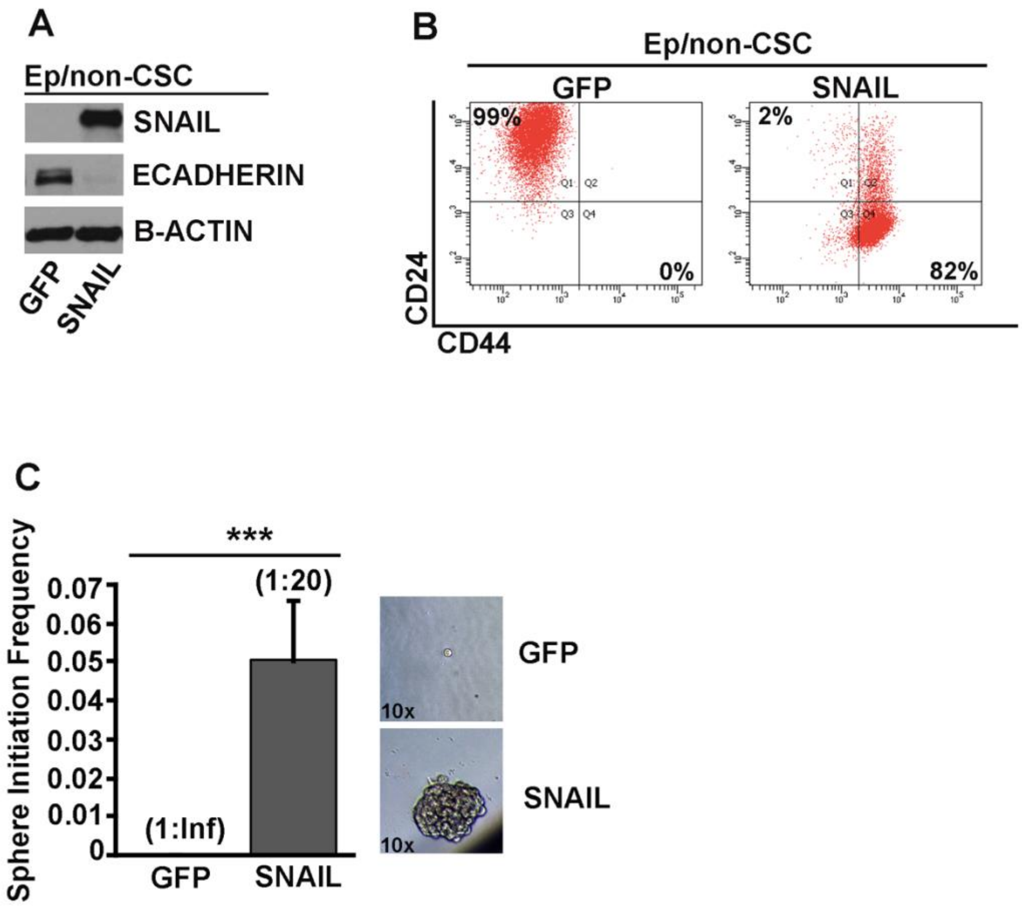
A
Ep/non-CSC
SNAIL
ECADHERIN
B-ACTIN
GFP
SNAIL
B
Ep/non-CSC
GFP
SNAIL
99%
0%
2%
82%
CD24
CD44
C
***
(1:20)
(1:Inf)
Sphere Initiation Frequency
0.07
0.06
0.05
0.04
0.03
0.02
0.01
0
GFP
SNAIL
10x
10x
GFP
SNAIL

SI Figure 6

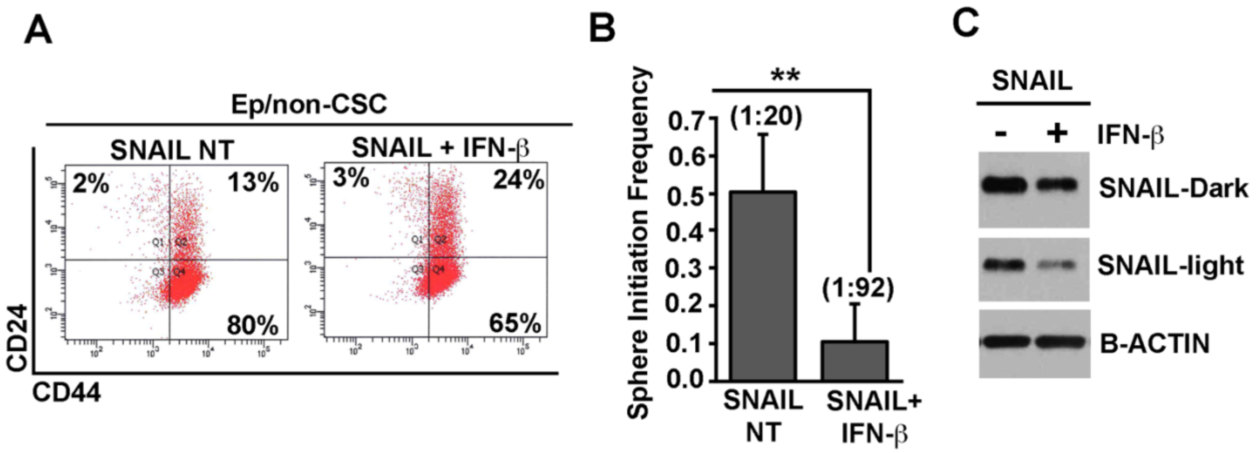
A
Ep/non-CSC
SNAIL NT
SNAIL + IFN-β
2%
13%
80%
3%
24%
65%
CD24
CD44
B
Sphere Initiation Frequency
**
(1:20)
(1:92)
0.7
0.6
0.5
0.4
0.3
0.2
0.1
0.0
SNAIL NT
SNAIL+ IFN-β
C
SNAIL
-
+
IFN-β
SNAIL-Dark
SNAIL-light
B-ACTIN

SI Figure 7

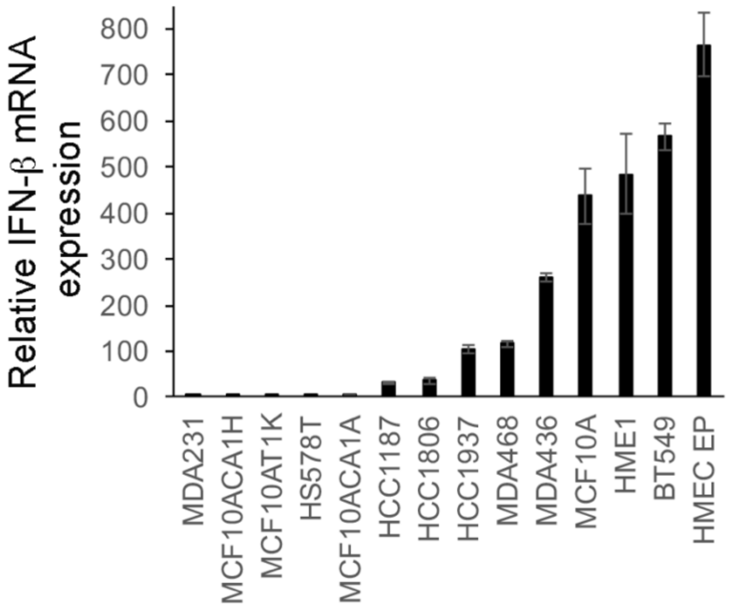
Relative IFN-β mRNA expression
800
700
600
500
400
300
200
100
0
MDA231
MCF10ACA1H
MCF10AT1K
HS578T
MCF10ACA1A
HCC1187
HCC1806
HCC1937
MDA468
MDA436
MCF10A
HME1
BT549
HMEC EP

SI Figure 8

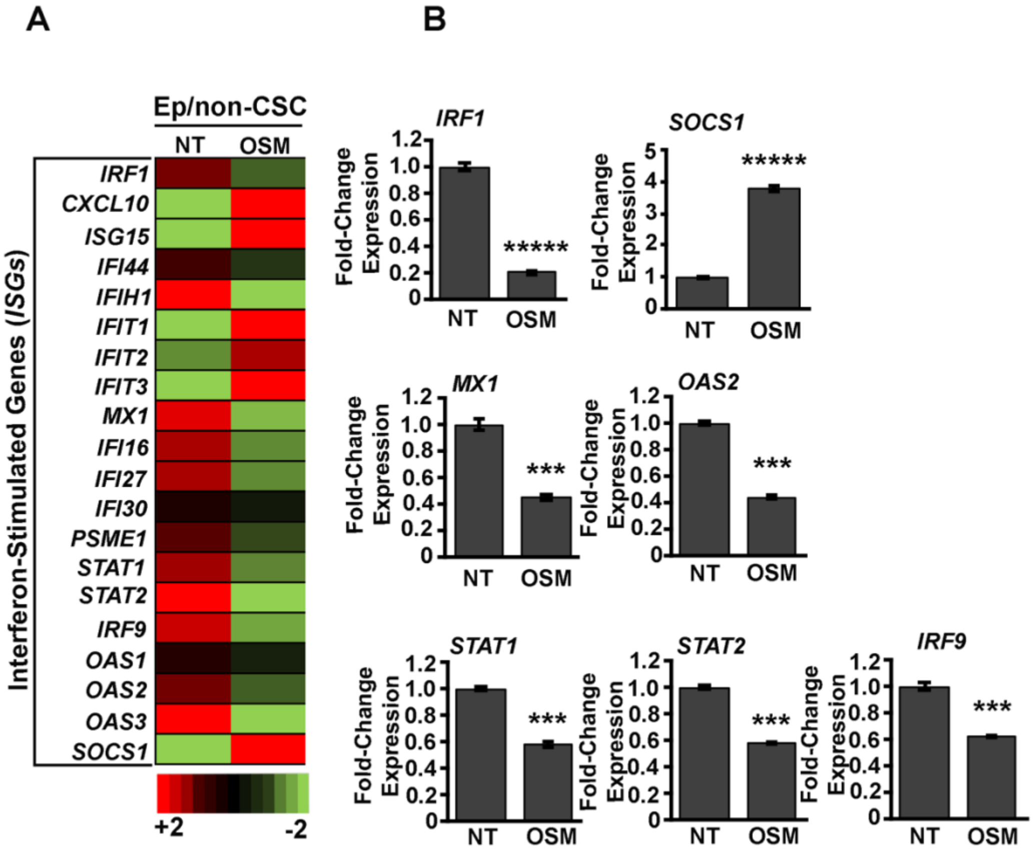
A
B
Ep/non-CSC
NT
OSM
Interferon-Stimulated Genes (ISGs)
IRF1
CXCL10
ISG15
IFI44
IFIH1
IFIT1
IFIT2
IFIT3
MX1
IFI16
IFI27
IFI30
PSME1
STAT1
STAT2
IRF9
OAS1
OAS2
OAS3
SOCS1
+2
-2
Fold-Change Expression
IRF1
SOCS1
MX1
OAS2
STAT1
STAT2
IRF9
*****
***

SI Figure 9

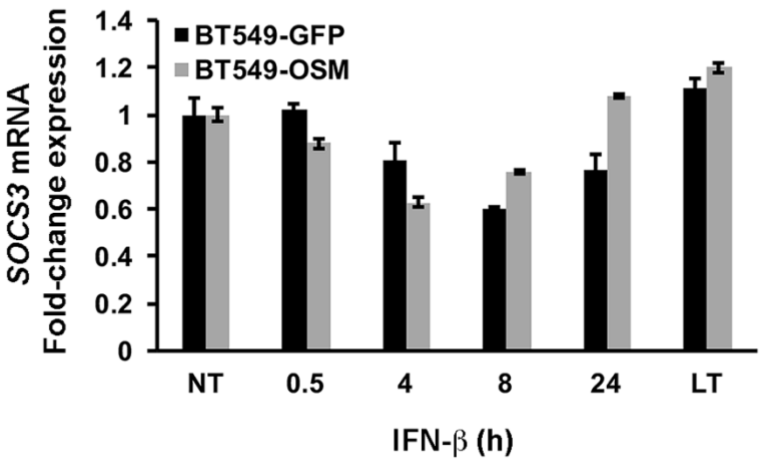
SOCS3 mRNA
Fold-change expression
1.4
1.2
1
0.8
0.6
0.4
0.2
0
BT549-GFP
BT549-OSM
NT
0.5
4
8
24
LT
IFN-β (h)

SI Figure 10

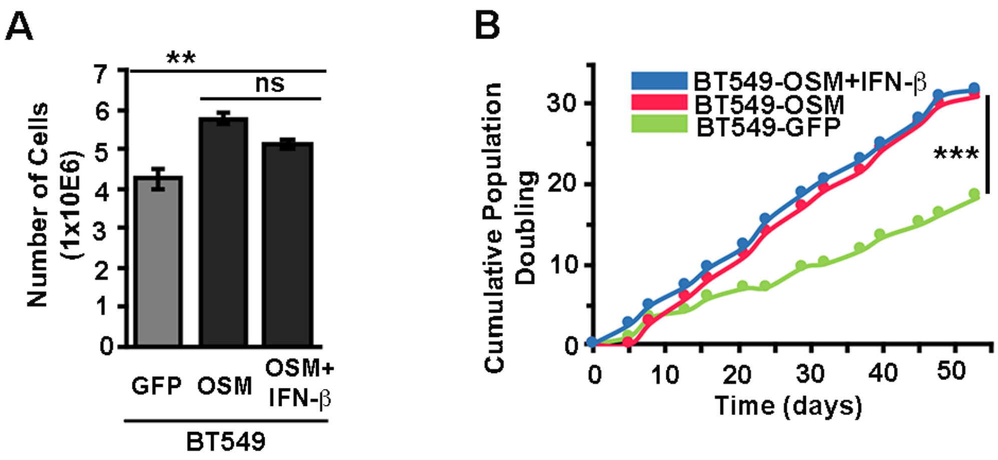
A
**
ns
Number of Cells (1x10E6)
7
6
5
4
3
2
1
0
GFP
OSM
OSM+ IFN-β
BT549
B
BT549-OSM+IFN-β
BT549-OSM
BT549-GFP
***
Cumulative Population Doubling
30
20
10
0
0
10
20
30
40
50
Time (days)

SI Figure 11

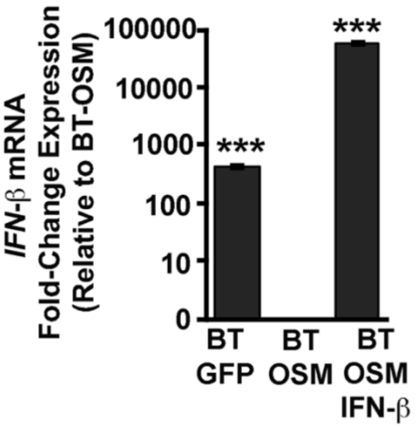
IFN-β mRNA
Fold-Change Expression
(Relative to BT-OSM)
100000
10000
1000
100
10
0
***
***
BT
GFP
BT
OSM
BT
OSM
IFN-β

SI Figure 12

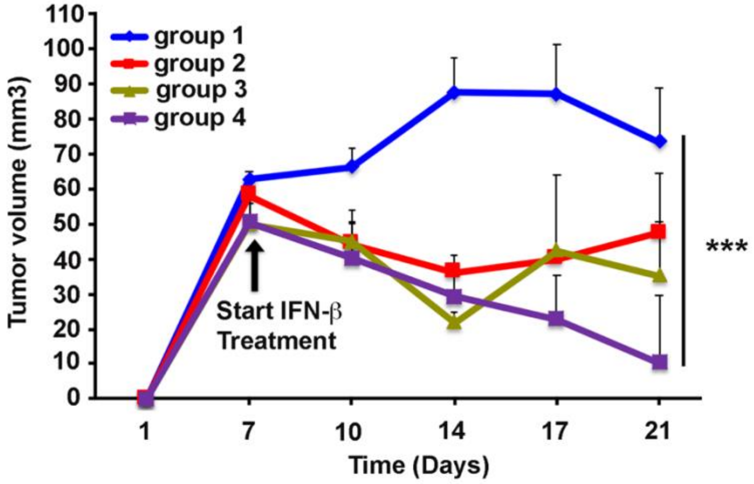

110
100
90
80
70
60
50
40
30
20
10
0
Tumor volume (mm3)
group 1
group 2
group 3
group 4
Start IFN-β
Treatment
***
1
7
10
14
17
21
Time (Days)

SI Table 1. Top 20 OSM target genes used in TNBC patient survival analysis.

Probe ID	Gene Symbol	OSM-induced gene expression
ILMN_1779234	CXCL6	4.5
ILMN_1787897	CXCL1	3.5
ILMN_1785732	TNFAIP6	3.0
ILMN_2347145	DCN	2.8
ILMN_1729801	S100A8	2.8
ILMN_1782538	VIM	2.8
ILMN_1666733	IL8	2.8
ILMN_1772910	GAS1	2.7
ILMN_3200140	LOC645638	2.6
ILMN_1782419	GNG11	2.5
ILMN_1657234	CCL20	2.5
ILMN_1772612	ANGPTL2	2.5
ILMN_2169152	SRGN	2.4
ILMN_1721770	PAPPA	2.3
ILMN_1792455	TMEM158	2.3
ILMN_2380237	C1QTNF1	2.2
ILMN_1813314	HIST1H2BK	2.2
ILMN_1805410	C15orf48	2.2
ILMN_181146	IRX3	2.2
ILMN_1785646	PMP22	2.2

The list of top 20 OSM-induced target genes was derived from our previously published microarray dataset (1) in which transformed HMEC-Ep/non-CSC were treated ± OSM (10 ng/mL) every 48h for 3 weeks. Gene expression is represented as Log2 fold-change (OSM treated relative to untreated control).

SI Table 2. Top 20 IFN-β target genes used in TNBC patient survival analysis.

Probe ID	Gene Symbol	IFN-β-induced gene expression
ILMN_2085862	SLC15A3	7.9
ILMN_2347798	IFI6	7.6
ILMN_1657871	RSAD2	7.1
ILMN_1788017	HSH2D	7.1
ILMN_2231928	MX2	6.3
ILMN_2370573	XAF1	4.6
ILMN_2388547	EPSTI1	4.3
ILMN_2054019	ISG15	4.2
ILMN_1759787	THBD	4.1
ILMN_1707695	IFIT1	4.0
ILMN_2173975	RTP4	3.8
ILMN_1795181	DDX60	3.8
ILMN_1654639	HERC6	3.7
ILMN_1760062	IFI44	3.4
ILMN_3243928	DDX60L	3.4
ILMN_1656670	HLA-G	2.2
ILMN_1800540	CD55	2.0
ILMN_1659913	ISG20	2.0
ILMN_2148785	GBP1	2.0
ILMN_2132458	CLDN4	2.0

The list of top 20 IFN-β-induced target genes were derived from our previously published microarray dataset (3) in which transformed HMEC-Ep/non-CSC were treated ± IFN-β (100 IU/mL) for 96h. Gene expression is represented as Log2 fold-change (IFN-β treated relative to untreated).

SI Table 3. Cells, reagents and source.

Cell line	Culture conditions	Source
HMEC-EP	MCDB170 + MM4 + growth factors supplementation (Junk et al.,2013; Garbe et al., 2009)	Engineered in Jackson Lab (Junk et al., 2013)
BT549	RPMI + 10% FBS + 0.023 IU/mL insulin	ATCC
MDA-MB-231<br>Hs578T<br>HCC1187<br>HCC1806<br>HCC1937	RPMI + 10% FBS	ATCC
MDA-MB-468	DMEM + 5% FBS (Cipriano et al., 2013)	ATCC
MCF10aT1K<br>MCF10a Ca1h<br>MCF10Ca1a	RPMI + 10% FBS	Dr. William Schiemann (Parvani et al., 2015)
MCF10a	RPMI + 10% FBS	ATCC
HME1	Medium 171 + MEGS (Cipriano et al., 2013)	Clontech

List of all cells used in the current study, their culture conditions and source from which they were purchased or where they were genetically engineered.
